# Harnessing naturally randomized transcription to infer regulatory relationships among genes

**DOI:** 10.1186/gb-2007-8-10-r219

**Published:** 2007-10-11

**Authors:** Lin S Chen, Frank Emmert-Streib, John D Storey

**Affiliations:** 1 Department of Biostatistics, University of Washington, 1705 NE Pacific St, Seattle, WA 98195, USA.; 2 Department of Genome Sciences, University of Washington, 1705 NE Pacific St, Seattle, WA 98195, USA.

## Abstract

We develop an approach utilizing randomized genotypes to rigorously infer causal regulatory relationships among genes at the transcriptional level, based on experiments in which genotyping and expression profiling are performed. This approach can be used to build transcriptional regulatory networks and to identify putative regulators of genes. We apply the method to an experiment in yeast, in which genes known to be in the same processes and functions are recovered in the resulting transcriptional regulatory network.

## Abstract 2

An approach is developed that utilizes randomized genotypes to rigorously infer causal regulatory relationships among genes at the transcriptional level. The approach is applied to an experiment in yeast, yielding new insights into the topology of the yeast transcriptional regulatory network.

## Background

It is now possible to measure DNA variation, RNA expression levels, and protein expression levels from thousands of genes in a given biologic sample [1-3]. Of great interest is inferring the 'wiring diagram', or the way in which many genes regulate one another and interact, from these sources of high-throughput data [4,5]. However, this goal is complicated by the fact that RNA levels, protein levels, phenotypes, and environmental conditions may all affect one another [6-10], creating intractable sources of confounding. This has made it difficult to distinguish correlation from causal regulatory effects, limiting the success and applicability of constructed genome-wide regulatory networks [11].

A number of integrative genomics studies have recently been conducted, in which large-scale genotyping and expression profiling is performed on individuals with randomized genetic backgrounds [12-15]. Typically, linkage analyses have been performed on these studies in order to detect quantitative trait loci (QTLs) underlying gene 'expression traits' [10,12-17]. Although these studies have shown that expression variation is highly heritable, this approach does not typically directly identify specific genes or mechanisms that are responsible for expression variation without additional experimentation. Instead of employing this experimental approach to genetically dissect expression traits, we have developed a method called 'Trigger' (Transcriptional Regulation Inference from Genetics of Gene ExpRession) for inferring causal regulatory relationships among all possible pairs of genes.

Randomization is the 'gold standard' for inferring causality of one variable on another [18-20]. This concept has successfully been applied in clinical trials to establish the causal effects of drugs on disease. Because DNA variation has a substantial and widespread effect on transcriptional variation [12-15,21-25], we show that randomizing DNA content provides a natural mechanism for randomizing RNA levels. By utilizing this randomization, we present a new theoretical result defining three testable conditions that, when true, imply that a directed causal relationship exists among a pair of transcripts, where this causal relationship is robust against confounding caused by hidden variables. Using this theoretical result, we develop a method to test directly for this causal relationship, which allows us to estimate the probability that the specific causal model is true. These probabilities can in turn be used to build meaningful regulatory networks, in which the certainty of any such network is easily quantified by the false discovery rate (FDR) [26]. In addition, the proposed approach explicitly identifies genes whose expression levels are responsible for variation of expression traits, overcoming a limitation of identifying only their QTLs.

The concept of causal modeling has previously been considered within the context of genetic variation [27-32]. Several of these existing approaches search for the best-fitting causal model among genes or traits linked to a common locus. The consideration of causality in those papers is justified by the joint linkage of traits to a common locus, thereby reducing the total number of causal models [29-31], but it is not justified by a randomization process. Whereas it has clearly been recognized that changes in linkage status when conditioning on traits in a specific order is strong evidence for a causal relationship among the traits [27,28,32], Trigger directly uses the 'Mendelian randomized' genotypes to test rigorously for causality. This allows for a strict definition of causality that can be directly tested. The proposed method has the notable feature that the test for causality is robust against false positives due to common hidden causal variables. The proposed method also provides a single significance measure for each potential causal relationship in such a way that they can be individually interpreted as well as combined to estimate an overall FDR of the network. Trigger avoids the ambiguities caused by selecting among several models by an often subjectively chosen model selection criterion.

We apply the proposed method to an experiment on yeast [12,33], in which two distinct strains were crossed to produce 112 independent recombinant segregant lines, and genome-wide genotyping and expression profiling were performed on each segregant line. Applying Trigger to this study yields genome-wide regulatory probabilities that can be used to construct networks with any desired FDR. We identify regulatory relationships among genes that recapitulate previous findings, provide new predictions, and yield new information about the topology of the yeast transcriptional regulatory network.

## Results and discussion

For an individual organism, DNA has the useful feature that it is usually a static variable, meaning that it is fixed and will not change with changing RNA levels, protein levels, phenotypes, or environmental conditions. By performing designed crosses of genetically distinct inbred or isogenic lines, one can randomize the genotypes of an organism from two or more genetic backgrounds, thereby producing independent realizations of DNA content from offspring to offspring [6]. At the same time, one may measure gene expression, or any other molecular or clinical phenotype of interest, on each resulting recombinant line.

**Figure 1:**
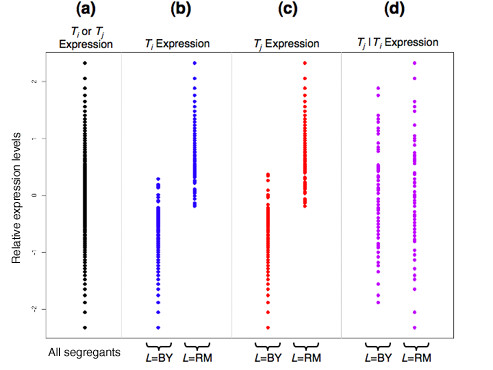
An illustration of the properties required to infer the causal relationship *L *→ *T*_*i *_→ *T*_*j*_. **(a) **All gene expression traits are normalized to follow a *N*(0,1) distribution. By the causality equivalence theorem, in order to conclude that *L *→ *T*_*i *_→ *T*_*j*_, it must be the case that **(b) ***T*_*i *_is linked to *L*, where the mean expression among segregants with allele at *L *inherited from the BY parental strain is different from the mean expression among segregants with allele at *L *inherited from the RM parental strain; **(c) ***T*_*j *_is also linked to *L*; and **(d) **the expression of *T*_*j *_given *T*_*i *_is no longer linked to *L*. Trigger is an algorithm to estimate the probability that all three conditions (shown in panels b to d) hold simultaneously.

We have developed Trigger as an approach for inferring regulatory relationships among all pairs of genes at the genome-wide level, based on these genetic cross experiments in which high-throughput expression profiling is also performed (Figure 1). However, one may also incorporate any other molecular or clinical phenotype of interest into the algorithm.

### Probabilities of transcriptional regulation

Suppose that there are *m *genes with transcription levels measured on recombinant offspring from an experimental genetic cross. (In the yeast experiment we consider, *m *= 6,216.) The goal is to use the data from such an experiment to estimate the probability that the transcription of gene *i *has a causal regulatory effect on the transcription of any other gene *j*, which we denote by *P*_*ij*_, where 'causal regulatory effect' means that a change in the transcription level of gene *i *results in a predictable change in the level of gene *j*. This is not necessarily through a direct molecular interaction; however, if we directly modulate the transcriptional level of gene *i*, then this should result in a corresponding change in the transcriptional level of gene *j*. Trigger provides a conservative estimate of these probabilities, denoted by ${\hat{P}}_{ij}$ for *i *= 1, ..., *m *and *j *= 1, ..., *m*.

**Figure 2:**
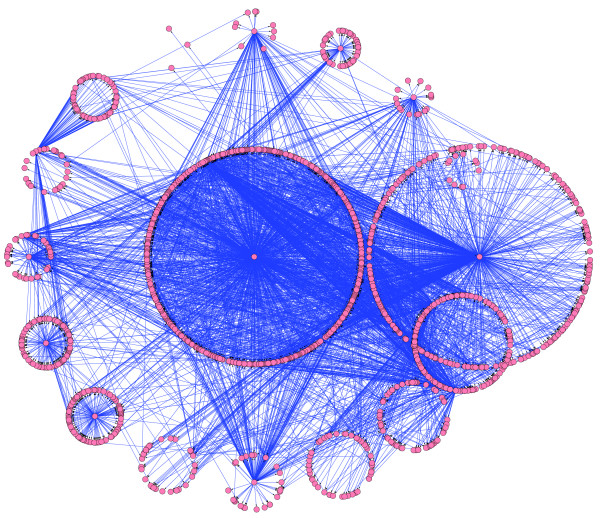
A transcriptional regulatory network drawn from a Trigger probability threshold of 90%. The network consists of 4,394 genes, 2,145 causal relationships, and 127 causal genes. Genes are represented by orange circles and causal relationships are represented by directed edges with black arrows.

These estimated regulatory probabilities can be used to build a regulatory network based on a directed graph. The probability that a directed edge exists from gene *i *to gene *j *in the network is estimated by ${\hat{P}}_{ij}$. One can directly threshold the entries, essentially setting those not meeting the threshold equal to zero. For example, one could remove all potential edges with ${\hat{P}}_{ij}$ < 90% while including those with ${\hat{P}}_{ij}$ ≥ 90%. Therefore, a directed edge would be drawn from gene *i *to gene *j *if and only if ${\hat{P}}_{ij}$ ≥ 90% (Figure 2). The resulting network has an easily quantified and interpretable FDR, and each directed edge has an estimated probability that it is true (see Materials and methods [below] and Additional data file 1).

In addition to constructing a regulatory network from these estimated probabilities, each gene *i *can be examined as a putative regulator, and hence a quantitative trait gene or 'quantitative trait transcript' [34]. Specifically, the probability that a specific gene *i *is a regulator for each other gene *j *is estimated as ${\hat{P}}_{ij}$. A threshold can be applied to these estimated probabilities to obtain the FDR of the significant genes (see Materials and methods [below] and Additional data file 1). This particular application of Trigger allows one to move beyond identifying QTL of expression traits to identifying a specific underlying causal quantitative trait transcript.

### Causal models of transcriptional regulation

Trigger is based on a rigorous mathematical framework that we developed for utilizing randomized genetic backgrounds and genome-wide expression in order to test rigorously for causality among transcription levels. The approach starts with a pair of transcripts and a locus to which both are linked. Let *L *be the locus, *T*_*i *_transcript *i*, and *T*_*j *_transcript *j*.

The goal is to identify triplets (*L*, *T*_*i*_, *T*_*j*_) such that *L *→ *T*_*i *_→ *T*_*j*_, where the arrow '→' means causation. The definition of 'causal' has been a topic of much interest [18,19]. Although definitions of causality differ slightly among the many articles published on this topic, in essence *T*_*i *_→ *T*_*j *_means that the ideal manipulation of *T*_*i *_will change the distribution of *T*_*j*_, whereas the ideal manipulation of *T*_*j *_will not disturb the distribution of *T*_*i*_. 'Ideal manipulation' of a variable means to change the variable in a manner that leaves every other variable unchanged, at the moment when the manipulation occurs [35]. This framework also applies to causality among random variables.

With the genetic cross experimental design, the genotype at a fixed locus *L *is a random variable, whose random outcome occurs before and independently from the subsequently measured expression values. For example, in the yeast experiment analyzed below, two haploid parental strains (BY and RM) were crossed to produce 112 recombinant haploid segregant strains. Because of the random segregation of chromosomes during meiosis, the inheritance of *L *= *BY *or *L *= *RM *is random. Therefore, when measuring the alleles at a single locus *L *across 112 segregants, we observe 112 genotypes being generated from some probability distribution. (See Materials and methods [below] for explicit details on the assumptions we make about the randomized genotypes among the loci.)

Because the randomization of *L *takes place before the expression levels of *T*_*i *_are measured, this implies that if *T*_*i *_is linked to locus *L *then *L *→ *T*_*i*_. This property is due to the well established principles in statistics showing that an association between two variables when one of them is properly randomized implies causation [19,20]. Additionally, the randomization of *L *is carried through to the variation in *T*_*i *_whenever *L *→ *T*_*i*_. If *L *→ *T*_*i*_, then segregants with *L *= BY have a different mean expression for *T*_*i *_than segregants with *L *= RM. Therefore, the randomization of *L *provides a randomization of the mean level of expression for *T*_*i*_. Figure 1a shows the transcriptional levels for a given gene, and Figure 1b shows a case in which it is linked to some locus *L*. Because the inherited allele *L *= BY or *L *= RM is random for each segregant, the mean level of expression for *T*_*i *_is random when *L *→ *T*_*i*_.

Importantly, some of the variation in *T*_*i *_will not be explained by *L*, specifically the random fluctuations of the transcription levels within each genotype (Figure 1b). Therefore, it is not possible to conclude that *T*_*i *_→ *T*_*j *_whenever *T*_*i *_and *T*_*j *_are significantly associated to *L*. This follows because there could be a common hidden variable affecting both *T*_*i *_and *T*_*j*_. (Note that if *T*_*i *_were perfectly randomized, then there would be no causal hidden variable for *T*_*i*_, which demonstrates the power of randomization.) Suppose that a hidden variable *H *is such that *H *→ *T*_*i *_and *H *→ *T*_*j*_. Because of this common hidden causal variable, any association between *T*_*i *_and *T*_*j *_would not allow us to conclude that *T*_*i *_→ *T*_*j *_even though *T*_*i *_has been partially randomized. In other words, the partial randomization of *T*_*i *_caused by *L *is now confounded by the effect of *H*. The common causal hidden variable *H *does not prevent *T*_*i *_→ *T*_*j *_from occurring; rather, we just are unable to draw any conclusion when this is the case, unless we are willing to model common hidden causal variables. Modeling common hidden causal variables has been shown to be particularly challenging in this high-dimensional setting [36], and doing so would require much additional work.

If there is a common causal hidden variable *H *that affects both *T*_*i *_and *T*_*j*_, then the Trigger method is designed to not make any conclusions about causality. However, if there is not a common hidden causal variable, then it is now possible, in a straightforward manner, to determine whether *T*_*i *_→ *T*_*j*_. The following new theorem identifies three conditions that are equivalent to the case in which both *L *→ *T*_*i *_→ *T*_*j *_and no common causal hidden variable affects both *T*_*i *_and *T*_*j*_. (See Materials and methods [below] for a mathematical proof.)

#### Causality equivalence theorem

The causal relationship *L *→ *T*_*i *_→ *T*_*j *_exists and there are no hidden variables causal for both *T*_*i *_and *T*_*j *_if and only if the following three conditions hold: *L *→ *T*_*i*_, *L *→ *T*_*j*_, and *L *⊥ *T*_*j *_| *T*_*i*_.

This theorem is used in the following manner. If *L *→ *T*_*i*_, *L *→ *T*_*j*_, and *L *⊥ *T*_*j *_| *T*_*i*_, then we may conclude that *L *→ *T*_*i *_→ *T*_*j *_exists and there are no hidden variables causal for both *T*_*i *_and *T*_*j*_. The fact that 'there are no hidden variables causal for both *T*_*i *_and *T*_*j*_' is not an assumption. Rather, it is a verified fact that follows when the three properties are true, as we show in the proof given in Materials and methods (below). We would prefer to detect all cases where *L *→ *T*_*i *_→ *T*_*j*_; however, as explained above, it is not yet possible to do so in the presence of common causal hidden variables.

Figure 1 provides a graphical representation of the three properties that must be satisfied. The last condition, *L *⊥ *T*_*j *_| *T*_*i*_, denotes that *T*_*j *_conditioned on the information in *T*_*i *_is independent from *L*. The first two conditions basically ensure that both transcripts are subjected to a common randomization. The third condition is the key one for inferring causality based on these randomizations. Basically, what the third condition determines is whether the causal effect from *L *on *T*_*j *_can entirely be captured by *T*_*i*_. If so, then *T*_*i *_is indeed a causal factor for variation in *T*_*j*_, with no hidden variables.

For computational and statistical efficiency, we limit *L *to be the locus of gene *i *(see Additional data file 1), which we denote as *L*_*i*_. We call *L*_*i *_→ *T*_*i *_the primary *cis *linkage and *L*_*i *_→ *T*_*j *_for any other gene *j *the 'secondary linkage' here. Because Pr(*T*_*i *_→ *T*_*j*_) ≥ Pr(*L *→ *T*_*i *_→ *T*_*j*_), we can obtain a conservative estimate of *P*_*ij *_by estimating Pr(*L *→ *T*_*i *_→ *T*_*j*_). From the causality equivalence theorem it follows that:

Pr(*L*_*i *_→ *T*_*i *_→ *T*_*j*_)

= Pr(*L*_*i *_→ *T*_*i *_and *L*_*i *_→ *T*_*j *_and *L*_*i *_⊥ *T*_*j *_| *T*_*i*_)

= Pr(*L*_*i *_→ *T*_*i*_) × Pr(*L*_*i *_→ *T*_*j *_| *L*_*i *_→ *T*_*i*_) × Pr(*L*_*i *_⊥ *T*_*j *_| *T*_*i *_| *L*_*i *_→ *T*_*i *_and *L*_*i *_→ *T*_*j*_)

The Trigger algorithm conservatively estimates *P*_*ij *_by estimating each probability in the above product from left to right and taking their product. (See Materials and methods [below] and Additional data file 1.)

### Application to yeast

We applied the Trigger algorithm to the yeast experiment (Materials and methods [below]) and found several interesting characteristics of the resulting regulatory probability matrix. Table 1 lists the overall significance results with different probability thresholds and Additional data file 2 contains the entire regulatory probability matrix. For example, at a probability threshold of 90%, we found 4,394 significant regulatory relationships among 2,145 genes where 127 are causal. Figure 2 shows a regulatory network drawn from the Trigger results at this threshold, where a directed edge is drawn from gene *i *to gene *j *if and only if *P*_*ij *_≥ 90%. It can be seen from Figure 2 that we have constructed a highly interconnected network where there is clearly a 'hub structure'.

**Table 1:** Overall significance of the regulatory probability matrix at different probability thresholds

Probability threshold	Number of putative regulators	Total number of genes	Number of edges	FDR (%)
0.95	76	1,075	1,499	2.7
0.90	127	2,145	4,394	6.0
0.85	194	3,150	8,826	9.4
0.80	255	4,044	15,448	12.9

FDR, false discovery rate.

**Figure 3:**
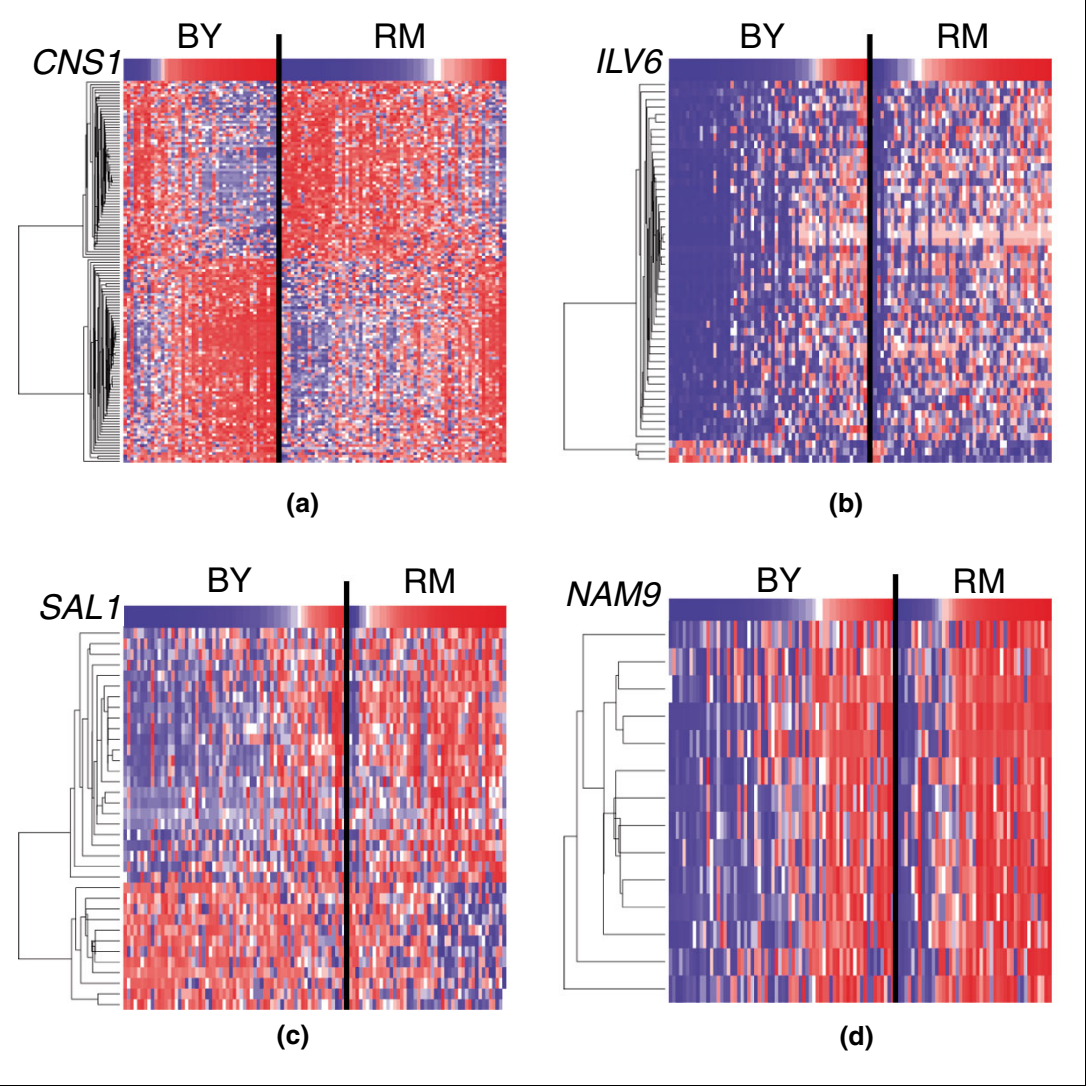
Heat-map display and hierarchical clustering of genes significantly regulated by the four putative regulators considered. The top row is the expression of the putative regulator (red indicates high expression, and blue low expression). All remaining rows are the hierarchically clustered significant genes. Each column represents a single segregant, where the segregants have been separated by genotype at the putative regulator's locus (black line). The columns have been ordered according to increasing expression of the putative regulator within each genotype. **(a) ***CNS1 *and its 144 significant genes. **(b) ***ILV6 *and its 51 significant genes. **(c) ***SAL1 *and its 36 significant genes. **(d) ***NAM9 *and its 14 significant genes.

We examined in detail four genes as putative regulators: *CNS1 *on chromosome 2, *ILV6 *on chromosome 3, *SAL1 *on chromosome 14, and *NAM9 *on chromosome 14. Each was highly significant for *cis *linkage, and the locus of each putative regulator had many significant secondary linking genes. At a 90% posterior probability cut-off (FDR = 6%), 144, 51 and 36 genes were significant for being regulated by *CNS1*, *ILV6*, and *SAL1*, respectively. At an 80% posterior probability cut-off (FDR = 11%), 14 genes were significant for being regulated by *NAM9*. The significant genes, posterior probabilities, and other relevant information for each putative regulator can be found in Additional data file 3. Note that each of these putative regulators is also a significant quantitative trait gene (or quantitative trait transcript) for each expression trait that it significantly regulates. Figure 3 shows heat maps of the four putative regulators and their corresponding significantly regulated genes. It can be seen that each significant gene is both linked to the locus of the putative regulator and has correlated expression with the regulator within each genotype, both of which are necessary but not sufficient for causality.

In order to determine whether the genes that are significant for each putative regulator show a coherent functional relationship, we employed the Gene Ontology (GO) database [37]. For each putative regulator, we queried the database among all significant genes and the regulator itself. This approach takes independently performed experiments and synthesizes the information obtained from those. The GO searches allowed us to test specifically whether common processes, functions, and components are present among each set of genes. Indeed, we found an abundance of significance for enriched GO terms for each set of genes corresponding to a putative regulator.

**Figure 4:**
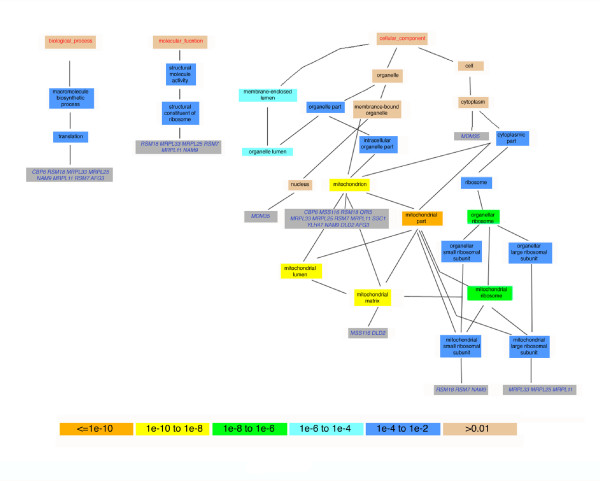
GO trees for *NAM9 *and 14 significantly regulated genes at 80% posterior probability threshold (FDR 11%). The colors of the boxes indicate the significance of the various Gene Ontology (GO) terms. *NAM9 *encodes a mitochondrial ribosomal component of the small subunit, involved in translation and mitochondrial small ribosome subunit [39-41]. Yeast is unviable under *NAM9 *deletion [38]. *NAM9 *is a structural constituent of ribosome, and it can be seen that seven out of the 14 genes, together with *NAM9*, are involved in translation. Five of them are also a ribosomal structural constituent and encode mitochondrial ribosomal subunits. Among the 14 putatively regulated genes, all except one uncharacterized gene are associated with mitochondria. FDR, false discovery rate.

Figure 4 shows the results of GO analysis for the putative regulator *NAM9*, which is a mitochondrial ribosomal component of the small subunit and inviable under deletion [38]. It is a structural constituent of ribosome, involved in translation and mitochondrial small ribosome subunit [39-41]. For the 14 genes significant at an 80% posterior probability threshold (FDR = 11%), 13 are known to be in the same or similar pathway as *NAM9*. The other significant gene is heretofore uncharacterized. Translation, structural constituent of ribosome, and mitochondrial small ribosome subunit are all highly significant terms in the GO tree.

Additional data file 1 (Figure S1) shows the results for the putative regulator *CNS1*, which is an essential tetratricopeptide repeat (TPR)-containing co-chaperone, deletion of which is inviable [42]. It binds both heat shock protein 82p (Hsp82p) and Ssa1p (Hsp70), and stimulates the ATPase activity of *SSA1*. *CNS1 *is involved in the protein binding process, and its cellular component is associated with cytoplasm [42-45]. Of the 144 genes significant at the 90% joint posterior probability cut-off (FDR = 6%), a substantial subset is involved in transferase activity and ribosome biogenesis and assembly, which coincides with the key role played by *CNS1 *in yeast. Many of the 144 genes were also found to be in the same pathway as *CNS1*; for example, *TRM8 *and *CNS1 *are both involved in a pathway for protein binding [46,47].

Additional data file 1 (Figure S2) shows the significant GO results for *ILV6 *and its 51 genes under statistically significant regulation. *ILV6 *is a regulatory subunit of acetolactate synthase, which catalyzes the first step of branched-chain amino acid biosynthesis [48,49]. Amino acid biosynthesis and its associated pathways are significantly enriched GO terms with *P *values below 10^-10^. Cyclohydrolase activity and lyase activity are some other significant pathways identified by GO analysis.

The putative regulator *SAL1 *is a probable transporter and a member of the calcium-binding subfamily of the mitochondrial carrier family, with two EF-hand motifs. It works in transporter activity and calcium ion binding [50], with its corresponding cellular component involved in the mitochondrial inner membrane [51]. From the GO analysis (Additional data file 1 [Figure S3]), we can see that a number of the 36 genes significantly regulated by *SAL1 *are associated with the mitochondrian and membrane GO terms. Six of the 36 significantly regulated genes are involved in mitochondrial inner membrane with high statistical significance (*P *< 10^-8^), a trend that is consistent with previous findings [50,51].

It should be noted that in the case of *SAL1 *no polymorphism exists in the immediate 500 base regions upstream or downstream of the *SAL1 *open reading frame. The linkage peaks occur approximately 13 kilobases and 21 kilobases on either side. This illustrates that linkage does not have to be due to an unequivocally *cis*-acting regulatory polymorphism in order for Trigger to work. On the contrary, there must simply be some locus to which both expression traits *T*_*i *_and *T*_*j *_are linked. We justified limiting the locus *L *to be in the 50 kilobases region of *T*_*i *_based on computational and statistical increases in efficiency (Additional data file 1).

In addition to these four well characterized putative regulators, we noticed that expression levels of a number of genes with relatively unknown function (for instance, *YSW1*, *PHM7*, and so on), were predicted to regulate a number of genes, with significant GO terms appearing for each set. Therefore, our results can potentially be used to predict properties of relatively unknown genes as well. Furthermore, several transcription factors significantly regulated a number of genes, including *HAP1 *[52,53] and *RAD16 *[54,55]. In previous work it was found that mutations in *GPA1 *and *AMN1 *lead to expression changes in genes whose expression exhibits linkage to each respective locus [14]. Missense mutations (leading to amino acid changes in the protein product) were identified in both *GPA1 *and *AMN1 *that appear to be the cause of the expression changes in the linking genes. In work to be reported in the future we examine the *GPA1 *and *AMN1 *cases in detail, showing that there appears to be common causal hidden variables involved. The Trigger approach is extended to take into account these common causal hidden variables, allowing us to recapitulate the previous findings regarding *GPA1 *and *AMN1*.

### Comparison with other approaches

#### Mendelian randomization

Recently, 'Mendelian randomization' was proposed as a technique in genetic epidemiology to study the environmental determinants of disease [27,28]. Trigger builds upon this concept in the sense that it also employs the randomization of genotypes as a starting point to infer causality. Essentially, we have extended this idea by deriving precise conditions under which the causality of one trait on another can be confirmed and by providing a statistical technique for estimating the probability that one trait is causal for another, among potentially thousands of traits.

#### Model selection approaches

The concepts of 'causality' and 'regulation' have been utilized in different ways in previous reports concerning the construction of biologic networks [29,30,32,56-60]. Among those using the more rigorous definition of causality [35,61], most published approaches have been to choose among the best fitting causal models by partial correlation or by model selection. The difference between our work and most previous work is that we explicitly test for and quantify each causal relationship of interest by using the randomization of genetic backgrounds built into the genetic cross experimental system. Furthermore, we assess the significance of each causal relationship by estimating the probability that the causal relationship is true, so that it can be considered in a straightforward manner with millions of other potential causal relationships.

**Figure 5:**
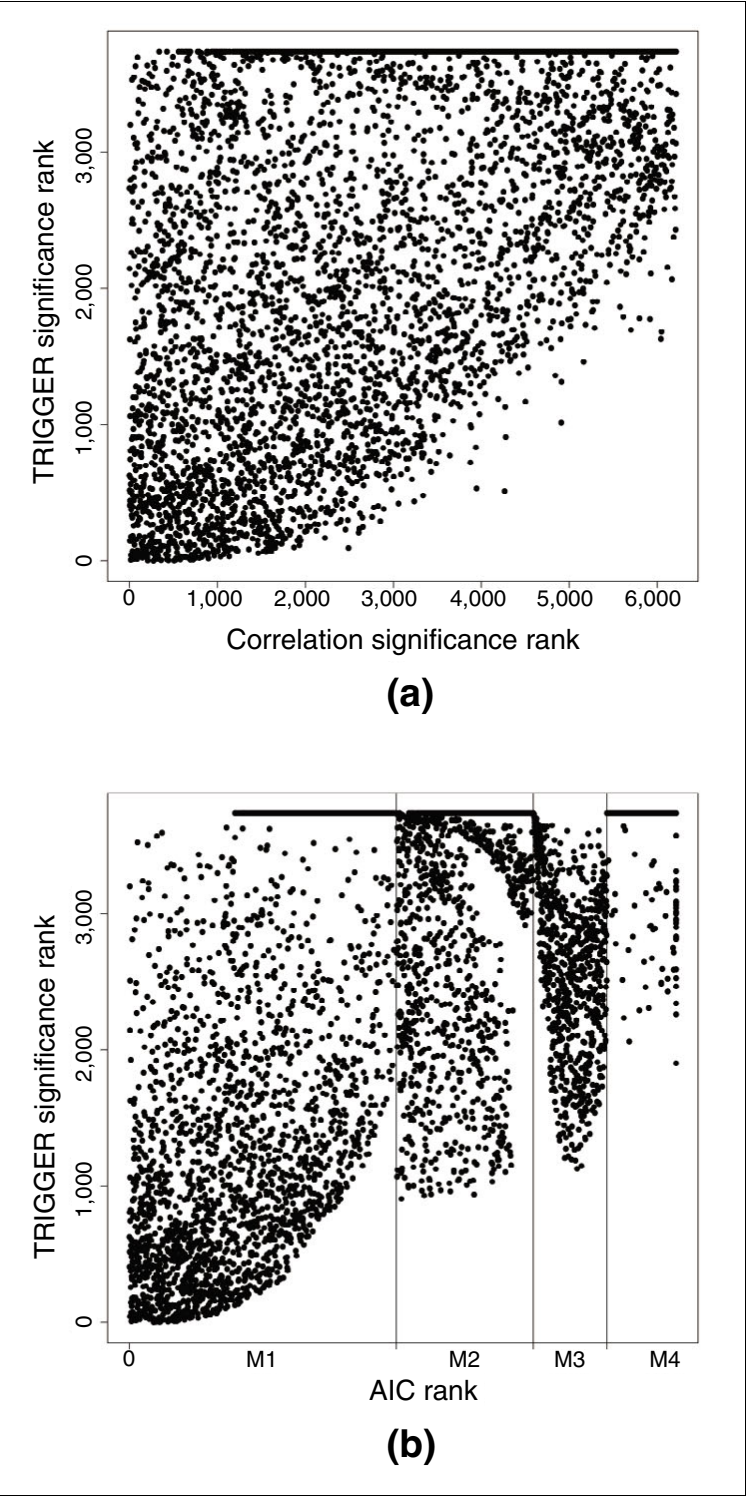
A comparison of Trigger with correlation and model selection for inferring existence causal relationship with *CNS1*. **(a) **Significance ranking according to Trigger versus the ranking according to correlation. Although this plot is not calculated conditional on linkage to the *CNS1 *locus, the plot conditional on linkage yields an equivalent qualitative conclusion. **(b) **Significance ranking according to Trigger versus the ranking according to model selection. For *CNS1 *and each gene, AIC was employed to selection among models capturing causality (M1), an inconclusive relationship (M2), linkage only (M3), and independence (M4). The x-axis is broken up into models M1 to M4; within each model type the genes were ranked according to their AIC score. For both correlation and model selection, it can be seen that there is not a strong relationship with Trigger in terms of the ranking, although a ranking in both is clearly necessary for a high Trigger probability. Note that many Trigger probabilities are zero, so the ranking does not extend all of the way to 6,216.

We have made some simple comparisons between Trigger and the model selection and correlation based approaches (Figure 5). In addition to Trigger showing different significance rankings relative to these approaches, it offers an increase in specificity. Most of the papers employing model selection have used the 'Akaike information criterion' (AIC) or derivatives thereof [29,31,32]. Among the about 38 million triplets (*L*_*i*_, *T*_*i*_, *T*_*j*_), the AIC model selection method [62] classifies about 15.4 million as causal, whereas Trigger identifies about 4,400 causal relationships with probability exceeding 90%. For the putative regulator *CNS1*, about 2,800 genes are classified as having a causal relationship with *CNS1 *by model selection, as opposed to the 144 Trigger found to be significant with probability exceeding 90%. The advantages that Trigger has over AIC and other model selection criteria are as follows: there is no generally applicable method to obtain an interpretable measure of significance based on these criteria (which is especially problematic when considering thousands of traits); and these approaches force one to model directly all possible hidden variables, making typically unverifiable assumptions about their underlying model [11].

### Extensions to other data types

We have presented Trigger within the context of inferring regulatory relationships based on gene expression data from organisms with randomized genetic backgrounds. However, this method may actually be applied to a much broader class of data types. Because the estimation is done in a nonparametric and scale-free manner (Materials and methods [below] and Additional data file 1), it is possible to combine any combination of expression, proteomic, metabolomic, and phenotypic data as the variables among which causal relationships are inferred. These may be considered separately or simultaneously, allowing one to discover regulatory relationships, say, among protein levels and transcriptions levels. The general requirement is that one must acquire organisms with random genetic backgrounds that are essentially stable as the expression levels and other potential traits are measured. The computational approach and statistical principles underlying the method remain the same for all of these data types.

## Conclusion

The Trigger algorithm allows one to infer transcriptional regulatory relationships among genes at the genome-wide level, based on experiments in which large-scale genotyping and expression profiling are performed among individuals with randomized genetic backgrounds. Moreover, the algorithm can be applied to any high-throughput phenotypic data in which genotypes or some other static regulatory mechanism has been randomized. Trigger works by identifying pairs of genes with expression levels both affected by a common randomized genotype and then testing for three key properties that we have mathematically demonstrated to be equivalent to a directed causal relationship among the pair of gene expression traits.

We applied Trigger to an experiment in yeast in which 112 independent recombinant segregants were subjected to genome-wide expression monitoring. The Trigger algorithm produced a regulatory probability matrix from this experiment that has been made available (Additional data file 2). This matrix can be used to build networks by a variety of techniques in which the noise level of any resulting network is easily assessed by the FDR. Our analysis of the results indicates that the proposed algorithm produces rich and biologically coherent information, mainly through a GO analysis of four putative regulators (*CNS1*, *ILV6*, *SAL1*, and *NAM9*).

Some caveats and limitations of the proposed approach are apparent. First, for any gene to be identified in a causal relationship, it must be linked to some locus. This is because the expression levels must be subjected to randomization based on the randomization of the genotypes. Therefore, this approach will not find all causal relationships. Second, a comprehensive genetic network requires additional measurements beyond transcriptional levels. Although it is straightforward to include all quantitative information in Trigger, such as transcription, protein, metabolite, and phenotype levels, it is not clear how to include important qualitative information, such as known protein interactions or transcription factor binding sites. The Trigger approach would have to be extended or combined with an existing approach to incorporate such data types.

The approach we have proposed is an early step toward moving beyond correlation and model selection based analyses of high-throughput molecular profiling data. Trigger offers a rigorous approach to inferring causality, based on the highly successful concept of randomized experiments, which has played a key role in science and medicine since its inception. This work also contributes to a better understanding of the ways in which multiple high-throughput data types can be combined to produce more informative estimates of the highly complex molecular networks underlying organisms.

## Materials and methods

### Expression measurements and genotyping

The expression and genotype data were recently reported elsewhere [12,33]. In that work, 112 segregants (one from each tetrad) were grown from a cross involving parental strains BY4716 (isogenic to the laboratory strain S288C) and the wild isolate RM11-1a. RNA was isolated and cDNA was hybridized to microarrays in the presence of the same BY reference material. Each array assayed 6216 yeast open reading frames. GeneChip Yeast Genome S98 microarrays were purchased from Affymetrix (Santa Clara, CA, USA). Genotyping was performed using GeneChip Yeast Genome S98 microarrays (Affymetrix) on all 112 F_1 _segregants. The resulting genetic map of 3,312 markers covered more than 99% of the genome.

### Assumptions regarding random genotypes

We simply point out here that the main assumption regarding random genotypes is that the *L*_*i *_are random variables occurring before and independently from the subsequently measured expression values. We also assume that the alleles inherited by different individuals at a fixed locus occurs independently; in other words, we assume that the crosses have been carried out independently. (If related segregants or offspring are collected, then Trigger can be adjusted to account for this.) However, we do not assume that the inheritance at several loci on a given chromosome occurs independently, and we make no other assumptions about independence of inheritance among loci. Segregation distortion, selection, and other traditionally problematic issues arising when performing genetic crosses for the purpose of genetic mapping do not invalidate Trigger.

As in all genetic crosses, the more independent the inheritance of the loci is, the more information there is in the experiment. For example, suppose that loci *L*_*i *_and *L*_*k *_are dependent (for instance, they are located on the same chromosome, or their segregation is dependent because of selection). Suppose also that L_i _→ T_i _→ T_j _and L_k _→ T_j_, but it is not the case that *L*_*k *_→ *T*_*i*_. Because *L*_*i *_and *L*_*k *_are dependent, it will not be the case that *L*_*i *_⊥ *T*_*j *_| *T*_*i*_, as not all linkage information for *T*_*j *_is captured by *T*_*i*_. Specifically, *L*_*i *_contains some information about *L*_*k *_because of their dependence, so *T*_*j *_| *T*_*i *_is not independent from *L*_*i*_. This is an example of how dependence of inheritance of different loci can reduce the power of Trigger. However, Trigger does not produce false positives because of this, so it is robust to linkage among loci on the same chromosome or other forms of dependence among loci.

### Proof of causality equivalence theorem

The proof of the theorem follows from well-established theory in graphical and causal modeling [35,61,63]. Several basic assumptions are typically made in causal modeling to avoid nonsensical situations. The 'causal Markov assumption' states that in a causal model, each variable is independent of all of its non-descendants given information about all of its direct causes. The 'faithfulness assumption' states that any conditional independence relationships in the population exist in the presence of the causal Markov assumption. Under the faithfulness assumption, conditional independence of two variables implies there is no direct edge between the two. Our proof also relies on the known result that if a hidden variable is causal for both *X *and *Y*, then the directed graph associated with *X *and *Y *can be represented by *X *→ *Y *[63].

We first show that if *L *→ *T*_*i *_→ *T*_*j *_with no hidden variables causal for both *T*_*i *_and *T*_*j*_, then *L *→ *T*_*i*_, *L *→ *T*_*j*_, and *L *⊥ *T*_*j *_| *T*_*i*_. Under these assumptions, the first two properties (*L *→ *T*_*i *_and *L *→ *T*_*j*_) are trivially true. Because there are no hidden variables involved, *T*_*i *_is the only direct cause of *T*_*j*_, and *L *is a non-descendant of *T*_*j*_, it follows by the causal Markov assumption that the third property (*L *⊥ *T*_*j *_| *T*_*i*_) holds.

We now show the more important direction of this equivalence: if *L *→ *T*_*i*_, *L *→ *T*_*j*_, and *L *⊥ *T*_*j *_| *T*_*i*_, then *L *→ *T*_*i *_→ *T*_*j *_and there are no hidden variables causal for both *T*_*i *_and *T*_*j*_. The third property (*L *⊥ *T*_*j *_| *T*_*i*_) implies that there is no direct edge between *L *and *T*_*j *_by the faithfulness assumption.

Let us first consider the case when there are no hidden variables causal for both *T*_*i *_and *T*_*j*_, so that the only variables involved in this causal graph are *L*, *T*_*i*_, and T_*j*_. Because of the second property (*L *→ *T*_*j*_), and there is no *direct *edge between *L *and *T*_*j*_, it must follow that there is a direct edge between *T*_*i *_and *T*_*j*_. Otherwise, *T*_*j *_is completely independent of *L*, which violates the second property. Thus, *L *→ *T*_*i *_- *T*_*j*_, where an edge without arrowheads implies dependence. If any two variables are dependent, then one is a cause of the other or there must be a third variable causal for both [63]. Thus, either *T*_*i *_is causal for *T*_*j*_, or *T*_*j *_is causal for *T*_*i*_, or both cases are true. *L *cannot be the common direct cause for both *T*_*i *_and *T*_*j*_, because no direct edge exists between *L *and *T*_*j*_. If *L *is an indirect cause of *T*_*j*_, then *T*_*i *_as the only other variable in the graph must be a direct cause of *T*_*j*_, implying that *T*_*i *_→ *T*_*j*_. If *T*_*j *_→ *T*_*i *_and the first property (*L *→ *T*_*i*_) holds, then it cannot be the case that the third property (*L *⊥ *T*_*j *_| *T*_*i*_) holds. Thus, *T*_*j *_is not causal for *T*_*i *_but it is true that *T*_*i *_→ *T*_*j*_, implying that *L *→ *T*_*i *_→ *T*_*j*_.

Now consider the second case in which there might be causal hidden variables in the graph. Because *L *is an independently randomized, static variable, there cannot be any hidden variables causal for both *L *and *T*_*i *_or both *L *and *T*_*j*_. The only possible existence of hidden causal variable in this graph is one affecting both *T*_*i *_and *T*_*j*_. However, if there is a common hidden cause for *T*_*i *_and *T*_*j*_, then *T*_*i *_→ *T*_*j *_[63]. If this is true, then *T*_*j *_| *T*_*i *_is dependent with *L*, contradicting the third property (*L T*_*j *_| *T*_*i*_). Therefore, *L *→ *T*_*i *_→ *T*_*j *_with no hidden variables affecting either of the two.

Note that it can be shown that the second and third properties (*L *→ *T*_*j *_and *L *⊥ *T*_*j *_| *T*_*i*_, respectively) imply the first property (*L *→ *T*_*i*_). However, we have designed Trigger to test for all three properties because conditioning on the first property increases the power to detect the state of the second and third properties.

Text

### Estimation of regulatory probabilities

The following method was developed to estimate the regulatory probabilities. Recall that by the causality equivalence theorem:

*P*_*ij *_= Pr(*L*_*i *_→ *T*_*i *_→ *T*_*j*_)

= Pr(*L*_*i *_→ *T*_*i*_) × Pr(*L*_*i *_→ *T*_*j *_| *L*_*i *_→ *T*_*i*_) × Pr(*L*_*i *_⊥ *T*_*j *_| *T*_*i *_| *L*_*i *_→ *T*_*i *_and *L*_*i *_→ *T*_*j*_)

To compute the joint posterior probability, the probabilities on the right hand side of the equation are estimated from left to right in that respective order. The basic algorithm works as follows (with specific details following) (Note that further details about steps 1 to 6 can be found in Additional data file 1.)

#### Step 1

Transform the expression data for each gene to follow a Normal distribution with mean 0 and variance 1.

#### Step 2

For each transcript, *T*_*i *_(*i *= 1, 2, ..., *m*), test the null hypothesis of no *cis *linkage to *L*_*i *_versus the alternative hypothesis of *cis *linkage to *L*_*i *_by performing a standard likelihood ratio test to obtain observed statistics *X*_*i *_(*i *= 1, 2, ..., *m*). Permute the expression data *B *times and perform the test on the permuted data to obtain null statistics $X_{i}^{0b}$ (*b *= 1, 2, ..., *B*). This is equivalent to testing *L*_*i *_→ *T*_*i*_.

#### Step 3

For each pair (*L*_*i*_, *T*_*i*_) from step 2, carry out the following. For all other transcripts *T*_*j *_(*j *≠ *i*), test the null hypothesis of no linkage to *L*_*i *_versus the alternative hypothesis of linkage to *L*_*i *_under the assumption that *L*_*i *_→ *T*_*i*_. Similarly to above, apply a standard likelihood ratio test to obtain observed statistics *Y*_*ij*_. Permute the expression data *B *times under the assumption that *L*_*i *_→ *T*_*i*_, and perform the test on the permuted data to obtain null statistics $Y_{ij}^{0b}$ (*b *= 1, 2, ..., *B*).

#### Step 4

For each triplet (*L*_*i*_, *T*_*i*_, *T*_*j*_), carry out the following. Estimate the conditional distribution of *T*_*j *_| *T*_*i*_, which is tractable under the Normal transformation. Test the null hypothesis of independence between *L*_*i *_and *T*_*j *_| *T*_*i *_versus the alternative hypothesis of dependence between *L*_*i *_and *T*_*j *_| *T*_*i*_. Again, apply a standard likelihood ratio test to obtain observed statistics *Z*_*ij *_for this test. Permute the expression data *B *times under the assumption that *L*_*i *_→ *T*_*i *_and *L*_*i *_→ *T*_*j*_, and perform the test on the permuted data to obtain null statistics $Z_{ij}^{0b}$ (*b *= 1, 2, ..., *B*).

#### Step 5

For each test from steps 2 to 4, the set of observed statistics and null statistics can be used to estimate the probability that the hypothesis of interest is true, based on previous methodology [17,26,64]. For example, the observed statistics *X*_*i *_(*i *= 1, 2, ..., *m*) and null statistics $X_{i}^{0b}$ (*i *= 1, 2, ..., *m*; *b *= 1, 2, ..., *B*) from step 2 can be used to form an empirical Bayes estimate of Pr(*L*_*i *_→ *T*_*i*_), which is equivalent to an estimate of the probability that the alternative hypothesis is true for each *i *= 1, 2, ..., *m*. The statistics from step 3 are used to estimate Pr(*L*_*i *_→ *T*_*j *_| *L*_*i *_→ *T*_*i*_), and the statistics from step 4 are used to estimate Pr(*L*_*i *_⊥ *T*_*j *_| *T*_*i *_| *L*_*i *_→ *T*_*i *_and *L*_*i *_→ *T*_*j*_).

#### Step 6

Multiply the three estimated probabilities together to get an estimate of *P*_*ij *_= Pr(*L*_*i *_→ *T*_*i *_→ *T*_*j*_), where:

$${\hat{P}}_{ij}=\hat{\text{P}}\text{r}(L_{i}\to T_{i})\times \hat{\text{P}}\text{r}(L_{i}\to T_{j}|L_{i}\to T_{i})\times \hat{\text{P}}\text{r}(L_{i}⊥T_{j}|T_{i}|L_{i}\to T_{i}\text{ and }L_{i}\to T_{j})$$

### False discovery rate estimation

A significance threshold can be applied to the probabilities for either the entire regulatory probability matrix or for a specific putative regulator. For the entire probability matrix, this would entail applying a threshold λ to the ${\hat{P}}_{ij}$, where we call *L*_*i *_→ *T*_*i *_→ *T*_*j *_significant if and only if ${\hat{P}}_{ij}$ ≥ λ. For a given putative regulator, the exact same thresholding would take place, except only the ${\hat{P}}_{ij}$ for a fixed putative regulator, gene *i*, would be considered. The estimate of the FDR corresponding to λ, FDR(λ), is as follows:

$$F\hat{D}R(\lambda )=\frac{\sum_{i,j}(1-{\hat{P}}_{ij})1({\hat{P}}_{ij}\geq \lambda )}{\#\{{\hat{P}}_{ij}\geq \lambda \}}$$

Where 1(${\hat{P}}_{ij}$ ≥ λ) is 1 or 0 according to whether ${\hat{P}}_{ij}$ ≥ λ or not, respectively, and # {${\hat{P}}_{ij}$ ≥ λ} is the total number of ${\hat{P}}_{ij}$ ≥ λ [17,65]. Further details and justification can be found in Additional data file 1.

## Abbreviations

FDR, false discovery rate; GO, Gene Ontology; Hsp, heat shock protein; QTL, quantitative trait locus; Trigger, Transcriptional Regulation Inference from Genetics of Gene ExpRession.

## Authors' contributions

LSC and JDS conceived the research, developed the methods, and wrote the paper. LSC analyzed the data. FES provided the visual organization of the network drawn in Figure 2.

## Additional data files

The following additional data are available with the online version of this paper. Additional data file 1 contains the supplementary text and figures. Additional data file 2 contains the entire matrix of regulatory probabilities for all genes, where the rows are genes acting as regulators and the columns are genes under regulation. Thus, the (*i*, *j*) entry of this matrix is the probability that the expression level of gene *i *is causal for the expression level of gene *j*. Additional data file 3 contains the list of significantly regulated genes, posterior probabilities, and other relevant information for each of the four putative regulators considered in detail.

## Supplementary Material

Additional data file 1 Presented are supplementary text and figures, as referenced in the main text. Click here for file

Additional data file 2 Presented is the entire matrix of regulatory probabilities for all genes, where the rows are genes acting as regulators and the columns are genes under regulation. Thus, the (*i*,*j*) entry of this matrix is the probability that the expression level of gene *i *is causal for the expression level of gene *j*. Click here for file

Additional data file 3 Presented is a list of significantly regulated genes, posterior probabilities, and other relevant information for each of the four putative regulators considered in detail. Click here for file

## References

[B1] Schena M, Shalon D, Davis RW, Brown PO (1995). Quantitative monitoring of gene expression patterns with a complementary DNA microarray.. Science.

[B2] MacBeath G, Schreiber SL (2000). Printing proteins as microarrays for high-throughput function determination.. Science.

[B3] Matsuzaki H, Dong S, Loi H, Di X, Liu G, Hubbell E, Law J, Berntsen T, Chadha M, Hui H (2004). Genotyping over 100,000 SNPs on a pair of oligonucleotide arrays.. Nat Methods.

[B4] Barabasi AL, Oltvai Z (2004). Network biology: Understanding the cell's functional organization.. Nat Rev Genet.

[B5] Ideker T (2004). Systems biology 101: what you need to know.. Nat Biotechnol.

[B6] Lynch M, Walsh B (1998). Genetics and Analysis of Quantitative Traits.

[B7] Weinzierl R (1999). Mechanisms of Gene Expression: Structure, Function and Evolution of the Basal Transcriptional Machinery.

[B8] Gasch AP, Spellman PT, Kao CM, Carmel-Harel O, Eisen MB, Storz G, Botstein D, Brown PO (2000). Genomic expression programs in the response of yeast cells to environmental changes.. Mol Biol Cell.

[B9] Lee TI, Rinaldi NJ, Robert F, Odom DT, Bar-Joseph Z, Gerber GK, Hannett NM, Harbison CR, Thompson CM (2002). Transcriptional regulatory networks in *Saccharomyces cerevisiae *.. Science.

[B10] Brem RB, Storey JD, Whittle J, Kruglyak L (2005). Genetic interactions between polymorphisms that affect gene expression in yeast.. Nature.

[B11] Chu TJ, Glymour C, Scheines R, Spirtes P (2003). A statistical problem for inference to regulatory structure from associations of gene expression measurements with microarrays.. Bioinformatics.

[B12] Brem RB, Yvert G, Clinton R, Kruglyak L (2002). Genetic dissection of transcriptional regulation in budding yeast.. Science.

[B13] Schadt EE, Monks SA, Drake TA, Lusis AJ, Che N, Colinayo V, Ruff TG, Milligan SB, Lamb JR, Cavet G (2003). Genetics of gene expression surveyed in maize, mouse, and man.. Nature.

[B14] Yvert G, Brem RB, Whittle J, Akey JM, Foss E, Smith EN, Mackelprang R, Kruglyak L (2003). *Trans*-acting regulatory variation in *Saccharomyces cerevisiae *and the role of transcription factors.. Nat Genet.

[B15] Cheung VG, Conlin LK, Weber TM, Arcaro M, Jen KY, Morley M, Spielman RS (2003). Natural variation in human gene expression assessed in lymphoblastoid cells.. Nat Genet.

[B16] Lan H, Stoehr JP, Nadler ST, Schueler KL, Yandell BS, Attie AD (2003). Dimension reduction for mapping mRNA abundance as quantitative traits.. Genetics.

[B17] Storey JD, Akey JM, Kruglyak L (2005). Multiple locus linkage analysis of genomewide expression in yeast.. PLoS Biology.

[B18] Rubin D (1974). Estimating causal effects of treatments in randomized and nonrandomized studies.. J Educ Psychol.

[B19] Holland P (1986). Statistics and Causal Inference.. J Am Stat Assoc.

[B20] Greenland S (1990). Randomization, statistics, and causal inference.. Epidemiology.

[B21] Cowles CR, Hirschhorn JN, Altshuler D, Lander ES (2002). Detection of regulatory variation in mouse genes.. Nat Genet.

[B22] Oleksiak MF, Churchill GA, Crawford DL (2002). Variation in gene expression within and among natural populations.. Nat Genet.

[B23] Jin W, Riley RM, Wolfinger RD, White KP, Passador-Gurgel G, Gibson G (2001). The contributions of sex, genotype and age to transcriptional variance in *Drosophila melanogaster*.. Nat Genet.

[B24] Yan H, Yuan W, Velculescu VE, Vogelstein B, Kinzler KW (2002). Allelic variation in human gene expression.. Science.

[B25] Rockman MV, Wray GA (2002). Abundant raw material for *cis*-regulatory evolution in humans.. Mol Biol Evol.

[B26] Storey JD, Tibshirani R (2003). Statistical significance for genome-wide studies.. Proc Natl Acad Sci USA.

[B27] Gray R, Wheatley K (1991). How to avoid bias when comparing bone marrow transplantation with chemotherapy.. Bone Marrow Transplant.

[B28] Smith GD, Ebrahim S (2003). 'Mendelian randomization': can genetic epidemiology contribute to understanding environmental determinants of disease?. Int J Epidemiol.

[B29] Schadt EE, Lamb J, Yang X, Zhu J, Edwards S, Guhathakurta D, Sieberts SK, Monks S, Reitman M, Zhang C (2005). An integrative genomics approach to infer causal associations between gene expression and disease.. Nat Genet.

[B30] Bing N, Hoeschele I (2005). Genetical genomics analysis of a yeast segregant population for transcription network inference.. Genetics.

[B31] Kulp D, Jagular M (2006). Causal inference of regulator-target pairs by gene mapping of expression phenotypes.. BMC Genomics.

[B32] Li R, Tsaih SW, Shockley K, Stylianou IM, Wergedal J, Paigen B, Churchill GA (2006). Structural model analysis of multiple quantitative traits.. PLoS Genetics.

[B33] Brem RB, Kruglyak L (2005). The landscape of genetic complexity across 5700 gene expression traits in yeast.. Proc Natl Acad Sci USA.

[B34] Passador-Gurgel G, Hsieh WP, Hunt P, Deighton N, Gibson G (2007). Quantitative trait transcripts for nicotine resistance in *Drosophila melanogaster *.. Nat Genet.

[B35] Spirtes P, Glymour C, Scheines R (2000). Causation, Prediction, and Search.

[B36] Spirtes P, Glymour C, Scheines R (2000). Constructing Bayesian network models of gene expression networks from microarray data. Proceedings of the Atlantic Symposium on Computational Biology, Genome Information Systems & Technology;.

[B37] Ashburner M, Ball CA, Blake JA, Botstein D, Butler H, Cherry JM, Davis AP, Dolinski K, Dwight SS, Eppig JT (2000). Gene ontology: Tool for the unification of biology. The Gene Ontology Consortium.. Nat Genet.

[B38] Steinmetz LM, Scharfe C, Deutschbauer AM, Mokranjac D, Herman ZS, Jones T, Chu AM, Giaever G, Prokisch H, Oefner PJ (2002). Systematic screen for human disease genes in yeast.. Nat Genet.

[B39] Boguta M, Dmochowska A, Borsuk P, Wrobel K, Gargouri A, Lazowska J, Slonimski PP, Szczesniak B, Kruszewska A (1992). NAM9 nuclear suppressor of mitochondrial ochre mutations in Saccharomyces cerevisiae codes for a protein homologous to S4 ribosomal proteins from chloroplasts, bacteria, and eucaryotes.. Mol Cell Biol.

[B40] Boguta M, Chacinska A, Murawski M, Szczesniak B (1997). Expression of the yeast NAM9 gene coding for mitochondrial ribosomal protein.. Acta Biochim Pol.

[B41] Biswas TK, Getz GS (1999). The single amino acid changes in the yeast mitochondrial S4 ribosomal protein cause temperature-sensitive defect in the accumulation of mitochondrial 15S rRNA.. Biochemistry.

[B42] Marsh JA, Kalton HM, Gaber RF (1998). Cns1 is an essential protein associated with the hsp90 chaperone complex in Saccharomyces cerevisiae that can restore cyclophilin 40-dependent functions in cpr7Delta cells.. Mol Cell Biol.

[B43] Dolinski KJ, Cardenas ME, Heitman J (1998). CNS1 encodes an essential p60/Sti1 homolog in *Saccharomyces cerevisiae *that suppresses cyclophilin 40 mutations and interacts with Hsp90.. Mol Cell Biol.

[B44] Nathan DF, Vos MH, Lindquist S (1999). Identification of SSF1, CNS1, and HCH1 as multicopy suppressors of a Saccharomyces cerevisiae Hsp90 loss-of-function mutation.. Proc Natl Acad Sci USA.

[B45] Hainzl O, Wegele H, Richter K, Buchner J (2004). Cns1 is an activator of the Ssa1 ATPase activity.. J Biol Chem.

[B46] Stoldt V, Rademacher F, Kehren V, Ernst JF, Pearce DA, Sherman F (1996). Review: the Cct eukaryotic chaperonin subunits of *Saccharomyces cerevisiae *and other yeasts.. Yeast.

[B47] Kim S, Willison KR, Horwich AL (1994). Cystosolic chaperonin subunits have a conserved ATPase domain but diverged polypeptide-binding domains.. Trends Biochem Sci.

[B48] Pang SS, Duggleby RG (1999). Expression, purification, characterization, and reconstitution of the large and small subunits of yeast acetohydroxyacid synthase.. Biochemistry.

[B49] Cullin C, Baudin-Baillieu A, Guillemet E, Ozier-Kalogeropoulos O (1996). Functional analysis of YCL09C: evidence for a role as the regulatory subunit of acetolactate synthase.. Yeast.

[B50] Chen XJ (2004). Sal1p, a calcium-dependent carrier protein that suppresses an essential cellular function associated with the Aac2 isoform of ADP/ATP translocase in *Saccharomyces cerevisiae *.. Genetics.

[B51] Belenkiy R, Haefele A, Eisen MB, Wohlrab H (2000). The yeast mitochondrial transport proteins: new sequences and consensus residues, lack of direct relation between consensus residues and transmembrane helices, expression patterns of the transport protein genes, and protein-protein interactions with other proteins.. Biochim Biophys Acta.

[B52] Pfeifer K, Kim KS, Kogan S, Guarente L (1989). Functional dissection and sequence of yeast HAP1 activator.. Cell.

[B53] Keng T (1992). HAP1 and ROX1 form a regulatory pathway in the repression of HEM13 transcription in *Saccharomyces cerevisiae *.. Mol Cell Biol.

[B54] Reed SH, You Z, Friedberg EC (1998). The yeast RAD7 and RAD16 genes are required for postincision events during nucleotide excision repair: in vitro and in vivo studies with rad7 and rad16 mutants and purification of a Rad7/Rad16-containing protein complex.. J Biol Chem.

[B55] Guzder SN, Sung P, Prakash L, Prakash S (1997). Yeast Rad7-Rad16 complex, specific for the nucleotide excision repair of the nontranscribed DNA strand, is an ATP-dependent DNA damage sensor.. J Biol Chem.

[B56] Weaver DC, Workman CT, Stormo GD (1999). Modeling regulatory networks with weight matrices.. Pac Symp Biocomput.

[B57] D'haeseleer P, Liang S, Somogyi R (2000). Genetic network inference: from co-expression clustering to reverse engineering.. Bioinformatics.

[B58] Friedman N, Linial M, Nachman I, Pe'er D (2000). Using Bayesian Networks to analyze expression data.. J Comput Biol.

[B59] Friedman N (2004). Inferring cellular networks using probabilistic graphical models.. Science.

[B60] Zhu J, Lum PY, Lamb J, GuhaThakurta D, Edwards SW, Thieringer R, Berger JP, Wu MS, Thompson J, Sachs AB (2004). An integrative genomics approach to the reconstruction of gene networks in segregating populations.. Cytogenet Genome Res.

[B61] Pearl J (2000). Causality: Models, Reasoning, and Inference.

[B62] Akaike H (1974). A new look at the statistical model identification.. IEEE Trans Automatic Control.

[B63] Richardson T, Spirtes P (2002). Ancestral graph Markov models.. Ann Stat.

[B64] Storey JD (2002). A direct approach to false discovery rates.. J Roy Stat Soc Ser B.

[B65] Newton MA, Noueiry A, Sarkar D, Ahlquist P (2004). Detecting differential gene expression with a semiparametric hierarchical mixture method.. Biostatistics.

[B66] Lehmann EL (1975). Nonparametrics: Statistical Methods Based on Ranks.

[B67] Lehmann EL (1986). Testing Statistical Hypotheses.

[B68] Anderson JA, Blair V (1982). Penalized maximum likelihood estimation in logistic regression and discrimination.. Biometrika.

