# Supplementary material for: Harnessing naturally randomized transcription to infer regulatory relationships among genes
Source: Genome Biol. 2007 Oct 11;8(10):R219. doi: 10.1186/gb-2007-8-10-r219 (PMC2246293; doi:10.1186/gb-2007-8-10-r219)
Supplement: Additional data file 1 — Presented are supplementary text and figures, as referenced in the main text. [file gb-2007-8-10-r219-S1.pdf]

Harnessing naturally randomized transcription to infer regulatory relationships among genes
Lin S. Chen, Frank Emmert-Streib, and John D. Storey
Additional Data File #1: Supplementary text and figures

Constructing Probabilistic Regulatory Networks

The probabilistic regulatory network constructed from the regulatory probabilities $\widehat{P}_{ij}$ would be composed of a set of genes as vertices, where any two genes that are connected are done so with a directed edge. The resulting network has an easily quantified and interpretable false discovery rate [26] (FDR) and each directed edge has an estimated probability that it is true.

The probability that a directed edge exists from gene i to gene j is estimated by $\widehat{P}_{ij}$. One can directly threshold these values, essentially setting those not meeting the threshold equal to zero. A directed edge is drawn from gene i to gene j if and only if $\widehat{P}_{ij} \geq \lambda$ for some threshold λ. For any given edge in the network, the probability that it is true is conservatively estimated by $\widehat{P}_{ij}$. The resulting FDR of the network (i.e., the proportion of edges in the network that are false positives) is estimated by $\sum(1 - \widehat{P}_{ij})/|G|$, where the sum is taken over all directed edges (i, j) exceeding the threshold and $|G|$ is the total number of edges in the estimated network (see *Detailed False Discovery Rate Estimation* below). The threshold in constructing such a network can be chosen to satisfy a desired FDR, a desired minimum edge probability, etc.

In addition to constructing a de-noised network from the regulatory probability matrix, subsets of the matrix can be extracted and thresholded similarly to examine putative regulators. In quantitative genetics parlance, a putative regulator significantly causal for other genes can be called a quantitative trait gene for these expression traits. To examine gene i as a putative regulator, all $\widehat{P}_{ij}$ would be examined for $j \neq i$. All genes j with $\widehat{P}_{ij} \geq \lambda$ are called statistically significantly regulated by gene i. The resulting FDR from this list of significant genes is estimated by $\sum(1 - \widehat{P}_{ij})/|S|$, where the sum is taken over j with $\widehat{P}_{ij}$ exceeding the threshold and $|S|$ is the total number of significant genes (see *Detailed False Discovery Rate Estimation* below). This extraction of genes can also be viewed as extracting a subset of the network, namely those genes under significant regulation from a chosen gene i.

Justification for Anchoring Trigger in cis-Linkage

The locus L that we consider when identifying cases of $L \to T_i \to T_j$ is the locus of the gene corresponding to T_i. That is, we test for linkage among markers within the region of T_i, sometimes referred to as *cis*-linkage. We make this restriction because the possible number of triplets (L, T_i, T_j) is on the order of several billion. The computational effort would be considerable to search over all triplets, while the great majority would not have the desired linkage present. It has been shown in several organisms that the proportion of expression traits showing *cis*-linkage is around 50% [9, 17], including in the yeast experiment examined here, which is much greater than the probability of an expression trait linking to a random locus. Therefore, by considering only those L at the locus of T_i we improve both the computational and statistical efficiency of the search.

Comparison of Trigger to Other Approaches

There are two basic approaches that have previously been proposed for building transcriptional regulatory networks [29, 30, 32, 56–60]. One is based on correlation or other distance functions that measure similarity in patterns of expression. For the *CNS1* putative regulator, we compared the significance ranking given by Trigger versus the ranking given by correlation and by model selection (Figure 5, main text). Figure 5a shows that many genes have highly correlated expression with *CNS1*, but do not appear to be regulated by *CNS1*. Thus, using correlation alone does not infer causality and it does not approximate the information given by Trigger.

The other approach is based on selecting the best causal model among a set of genes. The output of Trigger is essentially a Bayes network, in the sense that it is a probabilistic graphical model; however, the edges in the Trigger graph are directed and it allows for cycles. Rather than building a Bayes network by

fitting edges through partial correlations and/or model selection, we directly test for causality and quantify the certain of each directed edge.

In order to compare Trigger to the model selection approach, we considered all triplets (L_i, T_i, T_j) and chose the best model according to the "Akaike information criterion" (AIC) [62] . This was performed according to the Normal probability models employed in Trigger on the exact same transformed data. We chose among all possible models, similarly to previous work [29, 32]. We defined four classes of models: causal, inconclusive, linkage only, and independent. Among the ∼38 million triplets (L_i, T_i, T_j), the number occurring each class were 15.4 million, 3.6 million, 2.1 million, and 17.5 million, respectively. Thus, although Trigger finds ∼4400 significant causal relationships with probability exceeding 90%, one has to make use of 15.4 million claimed causal relationships when using the model selection approach. Figure 5b shows the model selection approach applied to the putative regulator *CNS1*. About 2800 genes are classified as having a causal relationship with *CNS1* by model selection, as opposed to the 144 Trigger found to be significant. It can be seen that all of the genes that are highly significant according to Trigger were classified as being causal according to model selection.

It is clear from the unwieldy number of genes found to be causal by model selection, that it has poor specificity. There is no straightforward way to assess the significance of all of these pairs of genes classified as being causal. The problem is even more complicated when using various *ad hoc* gene filtering techniques [29,58–60], because with each filtering step one selects false positives with favorable noise configurations that appear more and more like signal throughout the process. Given that all genes found to be causal by Trigger are captured by the model selection approach, it appears that Trigger provides the necessary specificity to build a network where the FDR is both estimable and truly low.

Further Details on the Estimation of Regulatory Probabilities

Specific details about Steps 1-6 of the algorithm to estimate the regulatory probabilities follow.

Step 1: Data normalization

Suppose that expression on n segregants has been collected. Let the expression data corresponding to transcript T_i be denoted by $t_{i1}, t_{i2}, \dots, t_{in}$. We transformed the expression data for each gene to standard Normal distribution with mean zero and variance one based on:

$$t^*_{ik} = \Phi^{-1}\left(\frac{rank(t_{ik})}{n+1}\right),\ k = 1, 2, \dots, n,$$

where $\Phi(\cdot)$ is the cumulative distribution function of $N(0, 1)$. This Normal transformation allowed us to easily characterize the conditional distributions needed to perform Steps 3 and 4, it made the data robust to outliers, and it allowed us to use parametric tests in a nonparametric fashion [66]. With very small n, this transformation may be problematic. The Trigger approach is most appropriate for studies with a large sample size, the yeast study with $n = 112$ about as small as we would recommend.

Step 2: Test for primary cis-linkage

Suppose that the measurements corresponding to L_i are $\ell_{i1}, \ell_{i2}, \dots, \ell_{in}$, where $\ell_{ik} = -1$ or 1 depending on whether the allele is inherited from the BY or RM strain, respectively. Because of the transformation, we used the following model to test if T_i is linked to L_i: $t_{ik} = \alpha_i + \beta_i \ell_{ik} + \epsilon_{ik}$, where ϵ_{ik} is independent random noise with a common variance for each gene, σ_i^2. [Note that although $t_{ik} \sim N(0, 1)$, this does not mean that ϵ_{ik} has a Normal distribution. However, for large n, this will be the case; also, we obtain our null statistics under permutations, so no assumption about ϵ_{ik} is necessary.] Under the null hypothesis of no linkage, $\beta_i = 0$. Under the alternative, $\beta_i \neq 0$. We performed standard generalized likelihood ratio test [67] and obtained the statistics X_i. Since there are a number of markers in the locus of gene i, the

marker that composed L_i is actually the one yielding the highest linkage within 50kb of the gene. In order to obtain the null statistics, X_i^{0b}, we simply replaced the t_{ij} with $t_{i,r(j)}$ and repeated the procedure, where $r(1), r(2), \ldots, r(n)$ is a random permutation of $1, 2, \ldots, n$.

Step 3: Test for secondary linkage

We model the relationship between T_j and L_i by $t_{jk} = \alpha_j + \beta_j \ell_{ik} + \epsilon_{ik}$. Therefore, T_j is linked to L_i if and only if $\beta_j \neq 0$. However, we perform the linkage test conditional that $L_i \to T_i$. The data corresponding to (T_i, T_j) jointly follow a bivariate normal distribution:

$$\begin{pmatrix} t_{ik} \\ t_{jk} \end{pmatrix} \sim N\left(\begin{pmatrix} 0 \\ 0 \end{pmatrix}, \begin{pmatrix} 1 & \sigma_{ij} \\ \sigma_{ij} & 1 \end{pmatrix} \right).$$

In order to condition on the scenario that T_i is linked to L_i, we need the joint distribution of the data corresponding to $(T_i|L_i, T_j|L_i)$:

$$\begin{pmatrix} t_{ik}|\ell_{ik} \\ t_{jk}|\ell_{ik} \end{pmatrix} \sim N\left(\begin{pmatrix} \alpha_i + \beta_i \ell_{ik} \\ \alpha_j + \beta_j \ell_{ik} \end{pmatrix}, \begin{pmatrix} \sigma_i^2 & \sigma'_{ij} \\ \sigma'_{ij} & \sigma_j^2 \end{pmatrix} \right).$$

The null hypothesis that T_j is not linked to L_i, given that $L_i \to T_i$ is equivalent to $\beta_j = 0$ (which also forces $\alpha_j = 0$). The alternative hypothesis is equivalent to $\beta_j \neq 0$. The observed statistics Y_{ij} are obtained by forming the standard generalized likelihood ratio statistic under these distributions. The null statistics Y_{ij}^{0b} are obtained by permuting the expression data similarly to the first test and recomputing the statistics.

Step 4: Test for conditional independence

It can be shown that a test of $T_j|T_i$ and L_i being independent is equivalent to a test of $(T_j - \rho_{ij} T_i)|T_i$ and L_i being independent, where ρ_{ij} is the correlation between T_i and T_j. Under the null hypothesis of independence it follows that

$$(t_{jk} - \rho_{ij} t_{ik})\,|t_{ik}, \ell_{ik} \sim N\left(0,\ 1 - \rho_{ij}^2\right).$$

Under the alternative,

$$(t_{jk} - \rho_{ij} t_{ik})\,|t_{ik}, \ell_{ik} \sim N\left(\mu_{ij}(\ell_{ik}),\ \sigma_{ij}^2(\ell_{ik})\right),$$

where $\mu_{ij}(\ell_{ik})$ and $\sigma_{ij}^2(\ell_{ik})$ are simply an allele specific mean and variance. The observed statistics Z_{ij} are obtained by forming the standard generalized likelihood ratio statistic under these distributions. The null statistics Z_{ij}^{0b} are obtained by permuting the expression data for genes i and j (applying separate permutations to each) and recomputing the statistics.

Step 5: Empirical Bayes probability estimation

The following procedure provides empirical Bayes posterior probability estimates for a set of observed and null statistics. Take, for example, the statistics from Step 2: observed statistics X_i and null statistics X_i^{0b}; $i = 1, 2, \ldots, m$, $b = 1, 2, \ldots, B$. We want to estimate from these the probability that the alternative is true for test i, for all $i = 1, 2, \ldots, m$. Assume that the average null probability density function (i.e., averaging over each true null test's density function) is g_0 and the average alternative density function is g_1. Then when considering all X_i together, a certain proportion, π_0 come from the null g_0 and the remaining proportion, $1 - \pi_0$, come from the alternative g_1. At the same time, all of the null statistics come from g_0. The probability that the alternative is true, $L_i \to T_i$, given X_i is:

$$\Pr(L_i \to T_i|X_i) = \frac{(1-\pi_0) g_1(X_i)}{\pi_0 g_0(X_i) + (1-\pi_0) g_1(X_i)} = 1 - \frac{\pi_0 g_0(X_i)}{\pi_0 g_0(X_i) + (1-\pi_0) g_1(X_i)}$$

Since the X_i come from $\pi_0 g_0 + (1-\pi_0)g_1$ and the X_i^{0b} come from g_0, these two sets of statistics can be combined to form an estimate of $g_0/[\pi_0 g_0 + (1-\pi_0)g_1]$, as has previously been proposed and applied [17,68]. The proportion of true nulls, π_0, is simply the FDR when calling all tests significant [64]. We estimate π_0 using well established methodology [26]. Applying this algorithm produces an estimate of these probabilities, $\widehat{\Pr}(L_i \to T_i | X_i)$, which we have written more simply as $\widehat{\Pr}(L_i \to T_i)$.

In order to estimate the probabilities $\Pr(L_i \to T_j | L_i \to T_i)$, we apply the same algorithm as above for each *fixed* i to the Y_{ij} and Y_{ij}^{0b}; $j \neq i$, $b = 1, 2, \ldots, B$. Thus for each fixed i, $\Pr(L_i \to T_j | L_i \to T_i)$ is estimated separately. This is because the priors associated with each (L_i, T_i) pair will be different.

Likewise, to estimate the $\Pr(L_i \perp T_j | T_i | L_i \to T_i \text{ and } L_i \to T_j)$, we apply the algorithm for each fixed i to the Z_{ij} and Z_{ij}^{0b}; $j \neq i$, $b = 1, 2, \ldots, B$. For this case, we estimate the probability that the null hypothesis is true, so estimation of the prior π_0 takes more care. Recall that when calculating this probability, we assume that $L_i \to T_i$ and $L_i \to T_j$. If we estimate π_0 among all statistics, then π_0 will be greatly overestimated because any case where T_j is not linked to L_i will trivially give the conditional independence. Let $\widehat{\pi}_0^{iY}$ be the prior estimate from the previous step; that is, $\widehat{\pi}_0^{iY}$ is an estimate of the proportion of expression traits secondary linked to L_i. Our goal is to estimate π_0^{iZ}, the prior probability of conditional independence for a fixed i, assuming that $L_i \to T_i$ and $L_i \to T_j$. To estimate π_0^{iZ} we take the $(1 - \widehat{\pi}_0^{iY})$ most significant proportion of T_j for secondary linkage from the previous step, and we use *only* their corresponding Z_{ij} and Z_{ij}^{0b} in above standard method [26] to estimate π_0^{iZ}.

A caveat is needed for the estimation of π_0^{iZ}. If we were able to identify the transcripts that are truly secondary linked and estimate π_0^{iZ} only on these, it is well known that the expected value of the resulting estimate is greater than or equal to the true π_0^{iZ}. This bias in the estimation of the prior is actually conservative for the estimates of $\Pr(L_i \to T_i)$ and $\Pr(L_i \to T_j | L_i \to T_i)$ because in those cases we are estimating the probability that the alternative hypothesis is true. However, for $\Pr(L_i \perp T_j | T_i | L_i \to T_i \text{ and } L_i \to T_j)$, we are estimating the probability that the null hypothesis is true. Therefore, the prior tends to be anti-conservative when the estimates are based only on the transcripts that are truly secondary linked. However, because $\widehat{\pi}_0^{iY}$ is conservative, we take a subset (on average) of the transcripts that are truly secondary linked, which biases the estimate back down.

In summary, the estimate of π_0^{iZ} is biased in both directions, and we have found that under likely scenarios it ends up as conservative. We performed simulations showing that for genes truly causal for many other genes, the estimation is conservative. For genes causal for very few other genes, the estimation may not conservative; however, in these cases the other probabilities tend to be small, so this bias has little effect. Therefore we believe the π_0^{iZ} estimate is reasonable at the moment, although an area of future work will be to resolve this more satisfactorily. The simulations presented below show that overall, the probability estimates have reasonably good behavior.

Remark on the permutations

The number of permutations performed for each of Steps 2, 3, and 4 do not have to be the same. Because of the data transformation, the permuted data for each transcript will be exchangeable. Thus it is possible to substantially reduce the number of necessary calculations by using the same set of permutation statistics for each fixed i in Steps 3 and 4. For Step 4, the null distribution does not depend on whether either transcript is linked to the locus, so the null distribution is the same for every pair of transcripts, making the computation of the null statistics more efficient.

Detailed False Discovery Rate Estimation

A significance threshold can be applied to the probabilities for either the entire regulatory probability matrix or for a specific putative regulator. For the entire probability matrix, this would entail applying a threshold λ to the $\widehat{P}_{ij}$ where we call $L_i \to T_i \to T_j$ significant if and only if $\widehat{P}_{ij} \geq \lambda$. For a given putative regulator, the exact same thresholding would take place, except only the $\widehat{P}_{ij}$ for a fixed putative regulator, gene i,

would be considered. Other biologically relevant subsets of genes may be considered, for example, those in a known pathway or those with a common GO annotation.

We define a "false discovery" to be any causal relationship $L_i \rightarrow T_i \rightarrow T_j$ called significant where in fact either (i) there is no causal relationship or (ii) there is a causal relationship, but both transcripts are affected by a hidden variable. The type (ii) false discovery is actually a biological true discovery, but we cannot mathematically distinguish this case from the type (i) false discovery. (See Simulation below, where we show that our estimation algorithm consistently controls the FDR of biological relevance.) The estimate of the FDR corresponding to λ, FDR(λ), is

$$\widehat{\text{FDR}}(\lambda) = \frac{\sum_{i,j} \left(1 - \widehat{P}_{ij}\right) 1\left(\widehat{P}_{ij} \geq \lambda\right)}{\#\left\{\widehat{P}_{ij} \geq \lambda\right\}},$$

where $1\left(\widehat{P}_{ij} \geq \lambda\right)$ is 1 or 0 according to whether $\widehat{P}_{ij} \geq \lambda$ or not, respectively, and $\#\left\{\widehat{P}_{ij} \geq \lambda\right\}$ is the total number of $\widehat{P}_{ij} \geq \lambda$. It is implicit that only (i, j) in the subset of interest are considered. The rationale and justification for this estimate has been described elsewhere [17]. The basic idea is that it has been shown that well behaved FDR estimates take the form of the ratio of the estimated expected number of false discoveries to the observed number of false discoveries. Here, $\sum \left(1 - \widehat{P}_{ij}\right) 1\left(\widehat{P}_{ij} \geq \lambda\right)$ serves as an estimated expected number of false discoveries. The probability causal relationship $L_i \rightarrow T_i \rightarrow T_j$ is a false discovery is $1 - P_{ij}$, which is also the expected number of false discoveries from this ordered pair. Thus, an estimate of the expected number of false discoveries is the sum of all $1 - \widehat{P}_{ij}$ that exceed the threshold.

Simulation Study

In order to assess the accuracy of Trigger, we simulated genetics of gene expression experiments from a mixture of six different models among the pairs of transcripts. Models 1-3 of Figure S4 are the three cases where there is a biologically relevant regulatory relationship. Model 1 is the specific case considered in the *Causality Equivalence Theorem*. In Model 2, there is also a hidden variable causal for both T_i and T_j. In Model 3, there is another genetic variant from the locus L that has a casual effect on T_j. Model 4 is a linkage only model; T_i and T_j are correlated only because they are both linked to L. Model 5 is a linkage model with hidden variables affecting both T_i and T_j. Models 4 and 5 do not include a regulatory effect from T_i to T_j. However, in these cases T_i and T_j are co-expressed, showing that co-expression of two transcripts may be completely unrelated to the regulation of one on another. Model 6 (not shown) is simply the random model, in which the expression of T_j is not linked to L and is also independent of T_i.

The experiments were simulated over a wide range of scenarios. Besides varying the relative abundance of Models 1-6, we also simulated different effect sizes for the linkages and the strength of regulation of one transcript on another. We present three scenarios, where one is representative of the best case behavior, another of the average behavior, and the third of worst case behavior. The main factor determining best case to average case to worst case is the proportion of transcripts truly regulated by T_i among all the transcripts T_j that are linked to the loci L. This is the prior probability in determining the probability that $L \perp T_j | T_i$ given $L \rightarrow T_i$ and $L \rightarrow T_j$.

We averaged the estimated FDR and the true FDRs for the full range of cut-offs of the $\widehat{P}_{ij}$ over 100 independent data sets for each scenario. As shown in Figure S5, the probability significance threshold is plotted against the FDRs for the three scenarios. The black line is the estimated FDR given by the proposed algorithm. Also plotted are two true FDRs. One is the true FDR according to our theory, which only counts the (L, T_i, T_j) from Model 1 as true discoveries. The other is the biological FDR, which counts all the (L, T_i, T_j) that have a regulatory effect $T_i \rightarrow T_j$, those in Models 1, 2 and 3. In the simulation where the prior of conditional independence is high (Figure S5a), the estimated FDR conservatively estimates both true FDRs.

In the scenario where the prior of conditional independence is low (Figure S5c), we sometimes overestimate this prior and consequently overestimate the joint posterior probabilities, resulting in an underestimated true FDR under the strict definition. However, in the yeast experiment, we found that when the prior of conditional independence was low, the probabilities of linkage were also low, which is not a relationship that we imposed in our simulation. For most loci with strong linkage among many transcripts, the estimated prior was around $40-70\%$, which is reflected in the average case scenario (Figure S5b). In those cases, the estimated FDR is sometimes slightly anti-conservative relative to the strict FDR when applying an extreme probability significance cutoff, but becomes conservative when a more moderate cutoff is chosen. Note that the estimated FDR is almost always conservatively estimated for the biological FDR in all three scenarios, which is the property we seek in practice. From this simulation, we conclude that Trigger provides reasonably well behaved significance estimates.

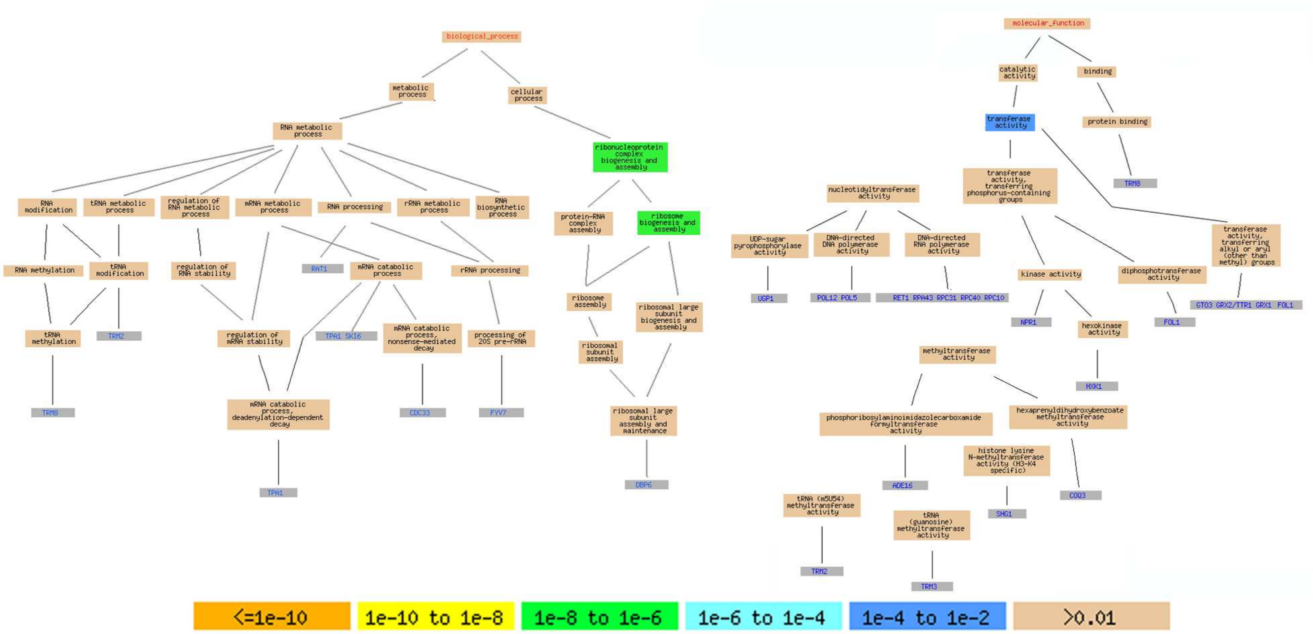

Figure S1: GO trees for *CNS1* and the 144 genes for which it is significantly causal. The colors of the boxes indicate the significance of the various GO terms. It can be seen that a significant proportion of the genes putatively regulated by *CNS1* are involved in ribosome biogenesis/assembly and transferase activity.

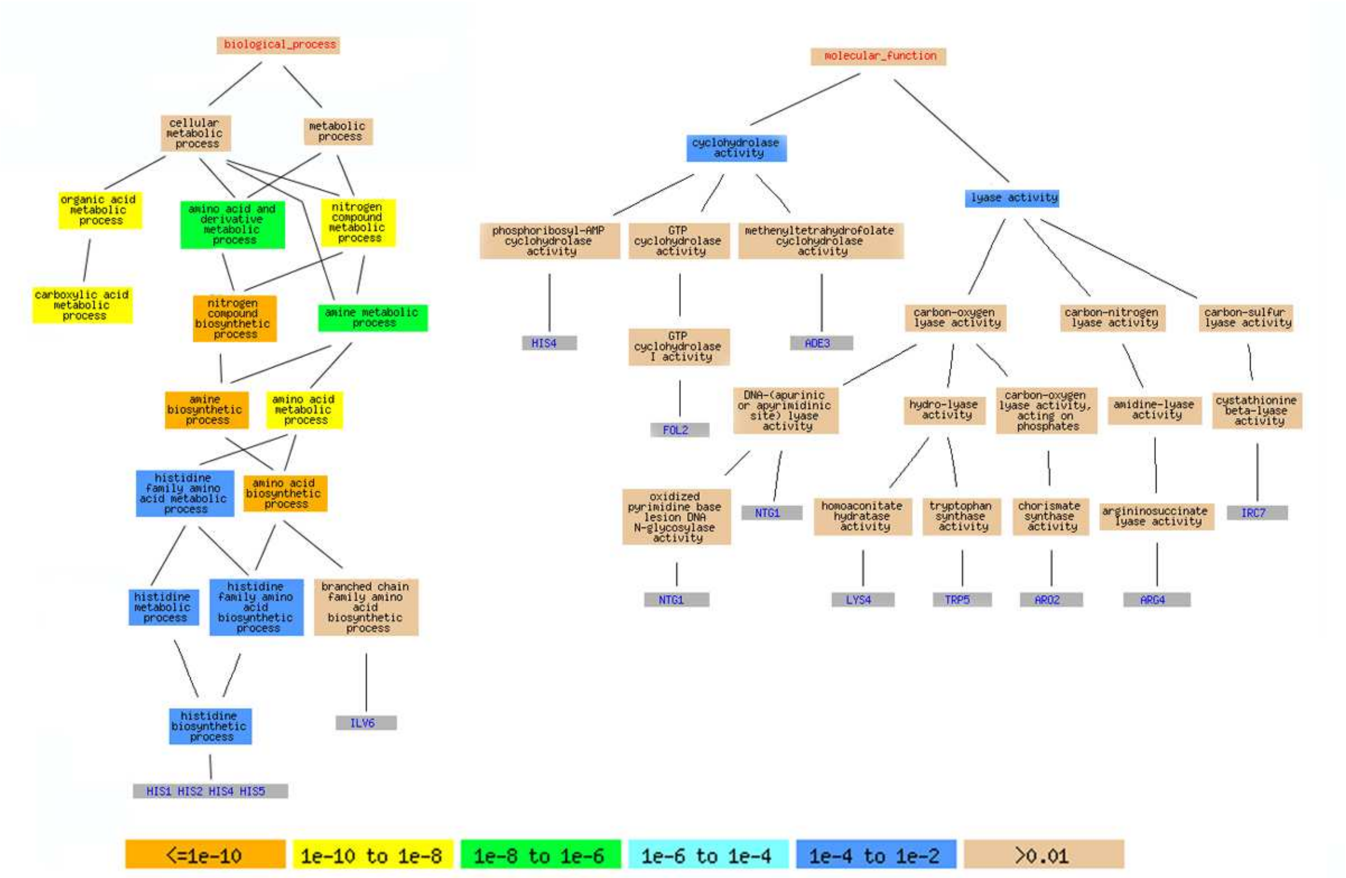

Figure S2: GO trees for *ILV6* and the 51 genes it significantly regulates. The colors of the boxes indicate the significance of the various GO terms. It can be seen that amino acid biosynthesis and its associated pathways are highly significant GO terms. Cyclohydrolase activity and lyase activity are also significant GO terms associated with *ILV6* and its regulated genes.

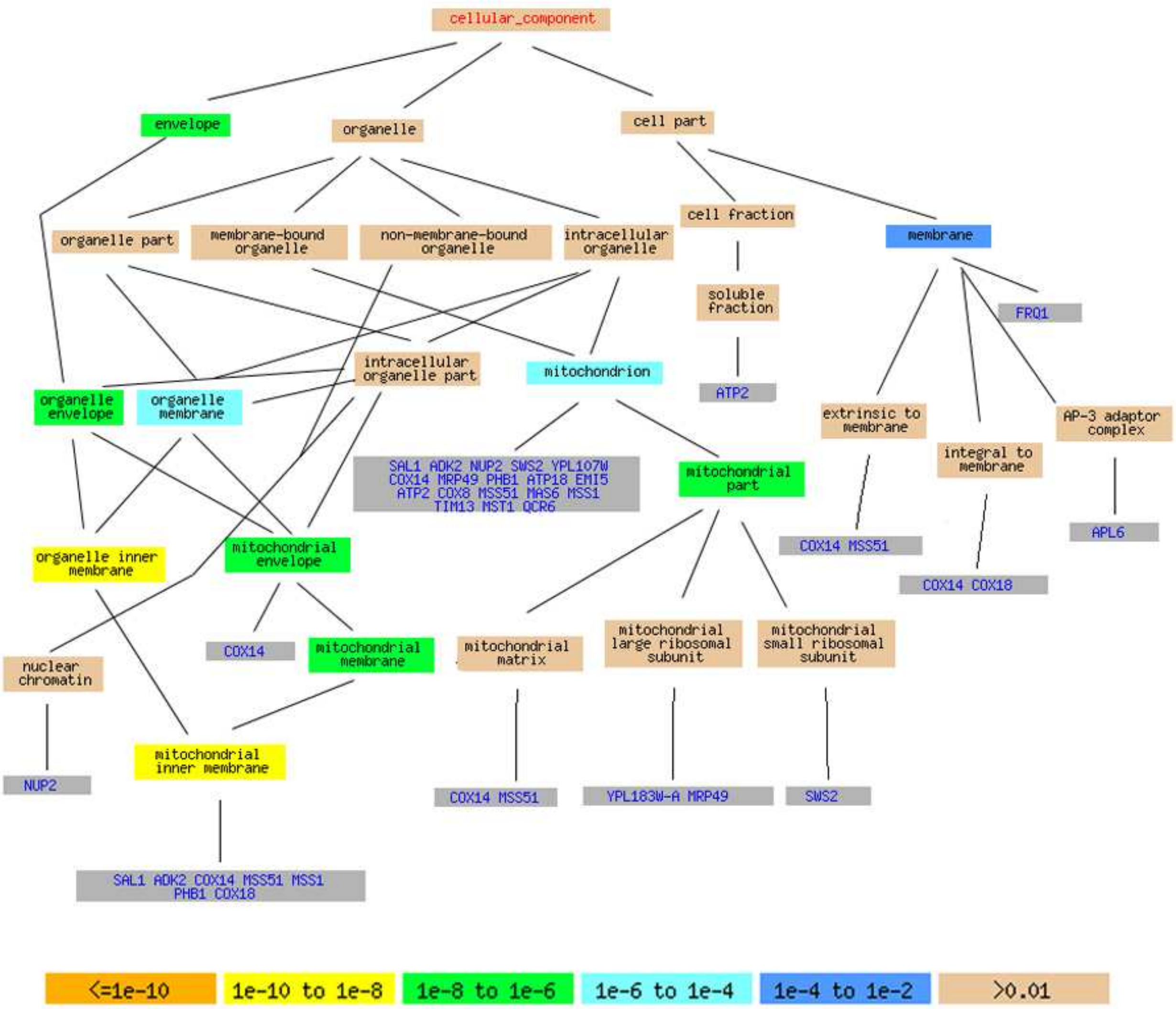

Figure S3: GO trees for *SAL1* and the 36 genes it significantly regulates. The colors of the boxes indicate the significance of the various GO terms. It can be seen that several of the genes are associated with mitochondria. Six of them are involved in the mitochondrial inner membrane with high significance.

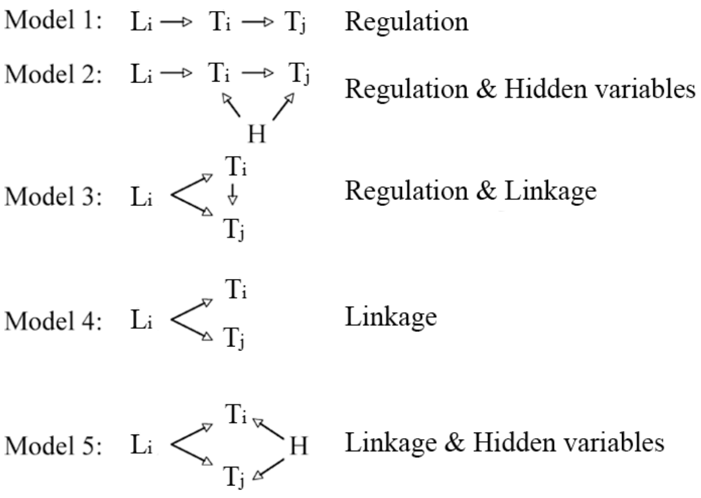

Figure S4: The five models employed in the simulation. A sixth model was also employed where T_j has no relationship to L or T_i.

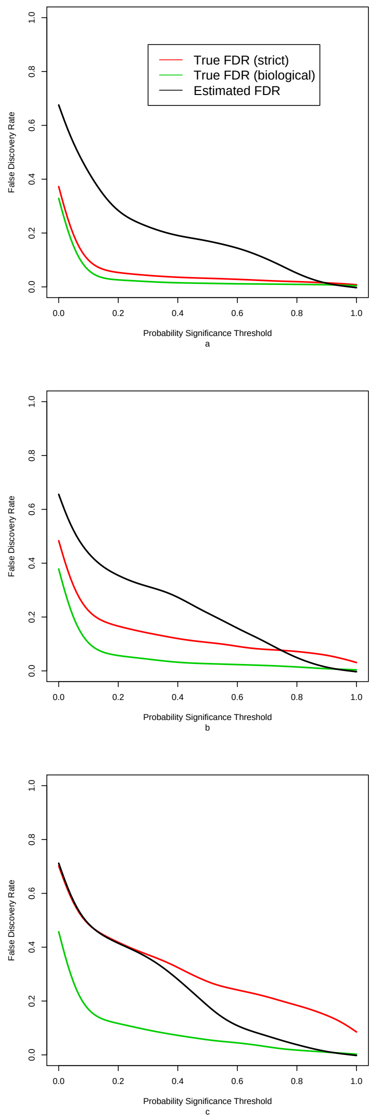

Figure S5: Plot assessing the accuracy of FDR estimation by Trigger with respect to two versions of a true FDR. The “strict” FDR (red line) is where a false discovery is defined to be any identified pair that does not exactly fit the criteria given in the *Causality Equivalence Theorem*. The “biological” FDR (green line) is where a false discovery is defined to be any identified pair where there is no causal relationship (this one allowing for biologically irrelevant violations in terms of hidden variables). The FDR versus the probability significant threshold is plotted for (a) a representative best case scenario, (b) a representative average case scenario, and (c) a representative worst case scenario. Whenever the estimated FDR (black line) is greater than or equal to a true FDR, the conclusion is that the Trigger procedure conservatively estimates the significance of the causal relationships.
