# Supplementary material for: Harnessing naturally randomized transcription to infer regulatory relationships among genes
Source: Genome Biol. 2007 Oct 11;8(10):R219. doi: 10.1186/gb-2007-8-10-r219 (PMC2246293; doi:10.1186/gb-2007-8-10-r219)
Supplement: Additional data file 3 — Presented is a list of significantly regulated genes, posterior probabilities, and other relevant information for each of the four putative regulators considered in detail. [file gb-2007-8-10-r219-S3.pdf]

Putative Regulator NAM9 Significant Genes

Significance Rank	Gene	Probability	Q-value
1	MDM35	0.9730355	0.0269645
2	CBP6	0.9688293	0.0290676
3	QRI5	0.96027	0.03262173
4	RSM18	0.9590218	0.03471085
5	RSM7	0.9535552	0.03705764
6	MRPL11	0.9247503	0.04342298
7	MRPL25	0.8878489	0.05324129
8	DLD2	0.8718405	0.06260606
9	YPR126C	0.8608533	0.07111058
10	MSS116	0.8493845	0.07906107
11	AFG3	0.8459413	0.08587904
12	SSC1	0.8396187	0.09208756
13	MRPL33	0.8109335	0.09954748
14	YPR125W	0.8073759	0.10619581

Putative Regulator CNS1 Significant Genes

Significance Rank	Gene	Probability	Q-value
1	UTP21	0.9994475	0.0005525
2	SHG1	0.9988951	0.0008287
3	TIF3	0.9971289	0.0015095
4	TTR1	0.9959549	0.0021434
5	RET1	0.995093	0.00269612
6	RAT1	0.9949243	0.00309272
7	NOP4	0.9946494	0.00341527
8	SSP1	0.994445	0.00368274
9	HXK1	0.9939922	0.00394108
10	RPC10	0.9936922	0.00417775
11	GRX1	0.9935405	0.00438518
12	NUG1	0.9930215	0.00460129
13	MAK11	0.9925364	0.00482147
14	RPA43	0.9924817	0.0050141
15	RCR2	0.9924766	0.00518139
16	GIS2	0.9903363	0.00546153
17	YOL079W	0.989947	0.00573162
18	FMP16	0.9888113	0.00603479
19	FOX2	0.9884966	0.00632261
20	YLR312C	0.9884178	0.00658559
21	CPR4	0.9872825	0.00687759
22	YER049W	0.9867505	0.00716722
23	DBP6	0.9865225	0.00744158
24	YLR257W	0.9860081	0.00771451
25	ADE16	0.9858675	0.00797123
26	NRP1	0.985739	0.00821314
27	GPT2	0.9847548	0.00847359
28	FAR8	0.9838864	0.00874645
29	YJL152W	0.9837764	0.00900428
30	RPL33A	0.98119	0.00933114

31	YER130C	0.978211	0.009733
32	CCT5	0.9778598	0.01012073
33	PEX18	0.9777114	0.01048945
34	NOG1	0.9766377	0.01086806
35	TRM2	0.9759846	0.0112437
36	PAM1	0.9757013	0.01160634
37	YLR198C	0.974296	0.01198736
38	YLR390W-A	0.9742184	0.01235037
39	VID28	0.9739524	0.01270158
40	MIS1	0.9737882	0.01303933
41	ATG15	0.9730006	0.01337982
42	NSA2	0.972684	0.01371164
43	NOP15	0.970913	0.0140692
44	KRR1	0.9704187	0.01442175
45	YVH1	0.9701227	0.01476521
46	SKI6	0.9688469	0.01512147
47	YPR003C	0.9683927	0.01547223
48	SAS3	0.9676192	0.01582449
49	SDP1	0.9665131	0.01618495
50	YER134C	0.9662664	0.01653592
51	TPK2	0.9652488	0.01689308
52	YAR1	0.9651343	0.01723871
53	IDH2	0.965089	0.01757215
54	CWC24	0.9637652	0.01791775
55	YDL025C	0.9616287	0.01828964
56	GTO3	0.959649	0.01868359
57	NHP2	0.9594152	0.01906782
58	PRB1	0.9593054	0.0194407
59	YGL085W	0.9591317	0.01980388
60	BAG7	0.9589434	0.02015809
61	FMP46	0.9582315	0.02051236
62	POL5	0.9572805	0.02087054
63	CHO1	0.9569014	0.02122336
64	YJL009W	0.9567304	0.02156784
65	MBR1	0.9561827	0.02191014
66	TRM8	0.9559761	0.02224519
67	RPC40	0.955881	0.02257167
68	RPT2	0.955288	0.02289726
69	ECM27	0.9540899	0.02323078
70	YGR066C	0.95342	0.02356434
71	SRP72	0.9530846	0.02389323
72	NSA1	0.9530214	0.02421386
73	RPS19A	0.9529996	0.024526
74	YGR054W	0.9521499	0.02484119
75	COQ3	0.9521312	0.02514823
76	PIR3	0.949467	0.02548224
77	RPC31	0.9486263	0.02581849
78	RHC18	0.9476626	0.02615848
79	SNF6	0.9471746	0.02649603

80	FRT2	0.9468327	0.02682942
81	GTR1	0.9467854	0.02715517
82	YLR241W	0.9458302	0.02748461
83	ISN1	0.9453406	0.02781202
84	SKG6	0.9447324	0.02813887
85	RFC2	0.944051	0.02846605
86	LAT1	0.9417796	0.02881203
87	RPL21B	0.9411149	0.0291577
88	PRS3	0.9405002	0.0295025
89	RPL18B	0.9399957	0.02984521
90	RPL39	0.9397566	0.03018297
91	YNR047W	0.9396092	0.03051492
92	FUN19	0.9391333	0.03084483
93	YDR063W	0.9389611	0.0311695
94	CDC33	0.9380756	0.03149668
95	YCR076C	0.9380241	0.03181752
96	RFC1	0.936979	0.03214255
97	SPC72	0.935851	0.03247252
98	FYV1	0.9353516	0.03280084
99	CDC123	0.9336571	0.03313965
100	CCP1	0.9334298	0.03347396
101	RTT107	0.9323259	0.03381257
102	RPL8B	0.9322256	0.03414553
103	YPL166W	0.9319484	0.03447471
104	YLR255C	0.9316051	0.03480087
105	TRM3	0.9296988	0.03513897
106	WRS1	0.9295896	0.03547172
107	RPL31B	0.9287154	0.03580642
108	CTK1	0.9261885	0.03615832
109	FRS1	0.925219	0.03651265
110	YIR016W	0.9245447	0.03686668
111	SPP41	0.9243202	0.03721635
112	GIS1	0.9240221	0.03756243
113	RPS28B	0.9233663	0.03790819
114	FYV7	0.92211	0.03825891
115	YBR280C	0.9219265	0.03860513
116	ZRG8	0.9205592	0.03895716
117	YMR193C-A	0.9205241	0.03930347
118	YNL303W	0.9202137	0.03964655
119	KRE33	0.9168577	0.04001206
120	YER158C	0.9154908	0.04038287
121	YGR287C	0.914994	0.04075165
122	YNL321W	0.914869	0.04111542
123	RIM101	0.9131835	0.04148697
124	NAF1	0.9127655	0.0418559
125	YER129W	0.9113745	0.04223006
126	UBA3	0.9103722	0.04260623
127	YLR177W	0.910172	0.04297806
128	YDR344C	0.908933	0.04335375

129	CUS1	0.9084033	0.04372773
130	FUN30	0.9082965	0.04409677
131	IML2	0.9080894	0.04446176
132	TAT1	0.9075302	0.04482546
133	NPR1	0.9070556	0.04518726
134	ROG3	0.9066712	0.04554652
135	FUR1	0.9059844	0.04590555
136	POL12	0.9044942	0.04627026
137	UGP1	0.9044358	0.04663007
138	CUL3	0.9043475	0.04698531
139	YPR1	0.903663	0.04734035
140	FOL1	0.9031641	0.04769389
141	EMI2	0.9016647	0.04805305
142	YHL039W	0.9012445	0.04841011
143	GLG1	0.9006153	0.04876658
144	RPL7A	0.90059	0.04911827

Putative Regulator ILV6 Significant Genes

Significance Rank	Gene	Probability	Q-value
1	TRP4	0.9991492	0.0008508
2	ARG2	0.9878409	0.00650495
3	YPL264C	0.9767701	0.01207993
4	GGC1	0.9513953	0.02121113
5	LYS4	0.9477482	0.02741926
6	NPR1	0.9467471	0.03172487
7	ASN1	0.9384877	0.03598021
8	CCP1	0.9371853	0.03933453
9	YKR015C	0.9281194	0.04295076
10	CPA2	0.927771	0.04587858
11	MCH1	0.927768	0.04827435
12	HIS4	0.9275132	0.05029205
13	TPN1	0.9274099	0.05200728
14	QDR3	0.927288	0.05348619
15	FRE6	0.9269955	0.05478741
16	UGA3	0.9268773	0.05593337
17	HIS5	0.9268326	0.05694714
18	SIP4	0.9268171	0.05784912
19	YPR059C	0.926398	0.05867822
20	YDR476C	0.9257965	0.05945448
21	YFR055W	0.9254836	0.06017172
22	ADE3	0.9246716	0.06086066
23	DMA2	0.924372	0.06150272
24	YMC1	0.922539	0.06216765
25	MUP3	0.9223266	0.06278788
26	HIS1	0.9219313	0.0633756
27	BNA1	0.9197716	0.06399978
28	VBA2	0.9192568	0.06459776
29	YIL056W	0.9185504	0.06517886
30	RIB3	0.918535	0.06572173

31	HIS2	0.9183132	0.06623673
32	ARG4	0.918257	0.0667213
33	YLR152C	0.918129	0.06718038
34	ARO2	0.916632	0.06765649
35	FOL2	0.9165337	0.0681082
36	NDT80	0.91596	0.06855075
37	SVL3	0.9154102	0.06898424
38	YOR203W	0.91517	0.06940123
39	CDC11	0.913822	0.0698314
40	SMF3	0.9130757	0.07025872
41	ISA1	0.9116642	0.07069963
42	YJL037W	0.9090397	0.07118203
43	ILM1	0.908625	0.07165163
44	TRP5	0.9074875	0.07212574
45	AAT1	0.9072789	0.07258342
46	CAN1	0.9058878	0.07305143
47	CLG1	0.9040486	0.07353867
48	NTG1	0.90318	0.07402369
49	MET30	0.902313	0.07450662
50	VHT1	0.901883	0.07497883
51	YGR110W	0.9004232	0.07546114

Putative Regulator SAL1 Significant Genes

Significance Rank	Gene	Probability	Q-value
1	COX18	0.998603	0.001397
2	EMI5	0.9984794	0.0014588
3	MSS1	0.9946793	0.0027461
4	USO1	0.9939644	0.00356848
5	YGL109W	0.9895113	0.00495252
6	SPT16	0.9892775	0.00591418
7	MSS51	0.9867727	0.00695891
8	ATP18	0.9867379	0.00774681
9	PIB1	0.9844101	0.00861827
10	APL6	0.9839595	0.00936049
11	ABP140	0.9838964	0.0099735
12	MAS6	0.9822315	0.01062308
13	SWS2	0.9618572	0.01273998
14	MST1	0.9584642	0.01479683
15	CDC8	0.9567195	0.01669574
16	UBC13	0.9556655	0.01842316
17	COX8	0.9482297	0.02038476
18	FRQ1	0.9433809	0.02239778
19	YPL183W-A	0.9373242	0.02451767
20	ARC1	0.9370305	0.02644027
21	QCR6	0.9362592	0.02821648
22	SCJ1	0.9343315	0.02991885
23	HAA1	0.9309623	0.03161967
24	YNG2	0.929491	0.03324005
25	MIH1	0.9283092	0.03477808

26	PHB1	0.9260392	0.03628511
27	MRP49	0.9243729	0.03774222
28	ADK2	0.9235757	0.03912373
29	YGL242C	0.9194337	0.04055278
30	NUP2	0.919357	0.04188912
31	YOR283W	0.9173502	0.04320398
32	DDC1	0.9172838	0.04443874
33	YPL107W	0.9170934	0.04560443
34	COX14	0.9151756	0.04675796
35	TIM13	0.904772	0.04814282
36	ATP2	0.9041175	0.04946892
